# Influence of Copigmentation on the Stability and Oxidative Stress of Anthocyanins from Purple Corn and Camu-Camu

**DOI:** 10.3390/molecules30234553

**Published:** 2025-11-26

**Authors:** Giulliano Nájera Bless, Victoria Muñoz Aguilar, Silvia Suarez-Cunza, Augusto Pumacahua-Ramos, Julio Santiago Contreras

**Affiliations:** 1 Departamento Académico de Química orgánica, Facultad de Química e Ingeniería Química, Universidad Nacional Mayor de San Marcos, Lima 15081, Peru; giulliano.najera@unmsm.edu.pe (G.N.B.); maria1305victoria@gmail.com (V.M.A.); 2 Departamento de Ciencias Dinámicas, Facultad de Medicina Humana, Instituto de Investigación de Bioquímica y Nutrición, Universidad Nacional Mayor de San Marcos, Lima 15001, Peru; ssuarezc@unmsm.edu.pe; 3 Departamento Académico de Ingeniería de Alimentos, Universidad Nacional Intercultural de Quillabamba, Quillabamba 08741, Peru; augusto.pumacahua@uniq.edu.pe

**Keywords:** anthocyanin, copigmentation, oxidative stress, purple corn, camu-camu, resveratrol, polyaspartic acid, TBARS

## Abstract

Alzheimer’s disease (AD) involves oxidative stress in brain tissue, creating a need for stable neuroprotective agents. Anthocyanins are potent antioxidants, but they are highly susceptible to degradation. This study evaluated anthocyanins from purple corn (PCA) and camu-camu (CCA) and the copigmentation effects of the phenolic acids cinnamic (C), ferulic acid (F), resveratrol (R) and polyaspartic acid (P) on the stability and neuroprotective capacity of these anthocyanins. HPLC-MS analysis identified twelve anthocyanins in PCA, including a new one (petunidin sophoroside). Copigmentation was assessed via UV-Vis spectroscopy, FTIR, TG, and stability tests at pH 7.4. The TBARS assay evaluated protection against oxidative stress in brain tissue. Copigment R provided the strongest hyperchromic effect for PCA, while P was most effective for CCA. TG analysis showed that copigmentation with R significantly improved the thermal and pH stability of both anthocyanin sources. All single copigmented samples showed improved stability of anthocyanins in physiological conditions. Furthermore, R-copigmented anthocyanins exhibited the most potent neuroprotective effects, significantly inhibiting lipid peroxidation. Thus, copigmentation, particularly with R, is a highly effective strategy for stabilizing anthocyanins and enhancing their potential as neuroprotective agents against oxidative stress.

## 1. Introduction

In recent years, there has been an alarming increase in the number of people suffering from AD [1]. Individuals with AD experience oxidative stress in brain tissue, leading to neuronal death [2,3]. In this context, the development of novel alternatives to protect the brain from oxidative stress would be highly beneficial for treating this condition. Anthocyanins are a subclass of water-soluble flavonoids. They are characterized by the presence of a flavylium ion in their structure and exhibit colors ranging from red to blue. Purple corn (*Zea mays* L.) and camu-camu (*Myrciaria dubia*) are plant species widely cultivated in Peru. Their anthocyanin-rich extracts exhibit promising antioxidant activities [4,5]. While cyanidin-3-glucoside is a major anthocyanin in both species, they possess distinct phytochemical profiles. Purple corn, in particular, exhibits a notably wider structural complexity, featuring diverse anthocyanin derivatives based on aglycones such as pelargonidin and peonidin, often with varying glycosylation and acylation patterns [6,7]. Despite their benefits, anthocyanins are highly susceptible to degradation due to pH or temperature changes. This sensitivity limits their applications [8].

Copigmentation is a phenomenon in which a non-covalent complex forms between a pigment (anthocyanin) and a copigment (typically colorless). This process results in changes in optical properties (hyperchromic and/or bathochromic effects), color intensification, and enhanced anthocyanin stability [9]. Previous studies have demonstrated that copigmentation of PCA combined with biopolymers such as alginate can significantly enhance their stability [10]. However, to the best of our knowledge, no similar copigmentation studies have been conducted on CCA.

The most common copigments are phenolic compounds, which facilitate π–π interactions and hydrogen bonding with anthocyanins [10]. R is a polyphenolic stilbene with strong antioxidant and neuroprotective activities. Its structure comprises two phenyl rings linked by a double bond; R exists as trans and cis isomers [11]. The planar R-trans isomer can interact with the flat region of the flavylium cation, favoring π-π stacking. This molecular fit can stabilize anthocyanins through face-to-face alignment, π-electron delocalization, and reduced vulnerability to water. Although R has been proposed as a candidate for anthocyanin copigmentation, key aspects such as its performance relative to classic phenolic acids and its impact on the bioactivity of the complexes are yet to be thoroughly investigated [12,13]. On the other hand, hydroxycinnamic acids such as ferulic acid and cinnamic acid are well-established copigments, extensively documented for their ability to enhance anthocyanin stability [14,15,16,17,18,19]. These compounds have also demonstrated promising biological activities relevant to AD, including antioxidant and neuroprotective effects, as well as the ability to inhibit amyloid-β aggregation. This dual functionality—as both stabilizers of anthocyanins and independent neuroprotective agents—makes them particularly attractive for developing multi-target therapeutic formulations aimed at AD [20,21,22].

Additionally, anthocyanins can also form non-covalent complexes with polymers to improve their stability [23]. P is a synthetic, water-soluble, non-toxic, and biodegradable polyamino acid. Structurally, P is a linear polymer composed of aspartic acid units in both L- and D-configurations, interconnected via α- and β-peptide bonds [24,25]. This unique combination of bond types and stereochemical configurations confers exceptional structural flexibility and a high density of ionizable carboxyl groups along the polymer backbone. The use of P as a copigment introduces a structurally novel class of stabilizer distinct from the polysaccharide-based polymers commonly employed, such as pectin, xanthan gum, alginate and chondroitin sulfate [26,27,28,29]. While these established polymers are composed of sugar monomer units, P has a protein-mimetic architecture. This allows it to engage with anthocyanins through a diverse set of interactions, similar to how natural proteins, such as β-lactoglobulin, function as copigments [30]. Additionally, P can exhibit a high negative charge density in aqueous solution at physiological pH, which is ideal for electrostatic interactions with positively charged molecules, such as anthocyanins in their flavylium cation form.

Previous studies have shown that copigmentation of anthocyanins can significantly improve their antioxidant activity [31,32]. Promisingly, copigmented anthocyanins have demonstrated an enhanced ability to mitigate oxidative stress, as evidenced by reduced markers of damage in serum and liver tissue in mice [16]. This suggests that the stabilized complexes may offer superior biological efficacy. Given that oxidative stress is a fundamental contributor to neurodegeneration in AD, we hypothesized that copigmented anthocyanins could provide enhanced protection to oxidative stress in brain tissues. Furthermore, previous studies have demonstrated that copigmented anthocyanins can inhibit acetylcholinesterase, a key enzymatic target in AD pharmacotherapy [33]. This evidence positions copigmentation as a promising strategy for developing multi-target AD therapeutics.

Therefore, this study aims to evaluate the effect of PCA and CCA copigmentation on stability under pH and temperature variations, as well as their protective capacity against oxidative stress in brain tissue. Known phenolic copigments—such as C, F, and R—were used, along with a novel polymeric copigment, P.

## 2. Results and Discussion

### 2.1. HPLC-MS Anthocyanin Characterization

Twelve distinct anthocyanins were identified by HPLC-MS analysis in the purple corn cob extract. Retention times and corresponding molecular ions for all detected anthocyanins are presented in Table 1.

**Figure 1:**
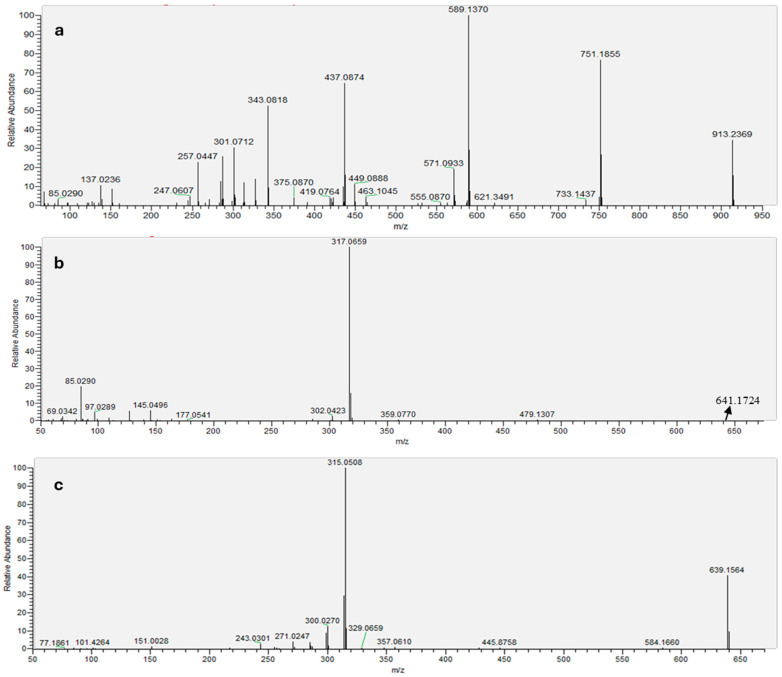
MS spectra of positive ion mode of catechin-(4,8)-peonidin 3,5-diglucoside (**a**); Positive ion mode of petunidin sophoroside (**b**) and negative ion mode of petunidin sophoroside (**c**).

Flavan-anthocyanins are minor constituents in some *Zea mays* species. While cyanidin-based derivatives are the most prevalent, those based on other aglycones, such as peonidin and pelargonidin, are considerably more scarce and have only been sporadically reported in this plant [34,35]. A previous study tentatively reported catechin-(4,8)-peonidin-3,5-diglucoside and catechin-(4,8)-pelargonidin-3,5-diglucoside in purple corn based on limited mass spectrometric data (two fragment ions: 751 and *m*/*z* 589, and *m*/*z* 721 and *m*/*z* 559) [36]. Our study provides a more detailed fragmentation analysis. As an example, we show the catechin-(4,8)-peonidin-3,5-diglucoside mass spectrum (Figure 1a). We confirm the identity of this compound through a comprehensive MS/MS spectrum that reveals three additional fragment ions (*m*/*z* 301, *m*/*z* 343, *m*/*z* 437) corresponding to the catechin moiety fragmentation in positive ion mode. This fragmentation profile corroborates the initial finding and provides structural validation of both the peonidin-3,5-diglucoside core and the interflavan linkage, offering a robust reference for future identifications.

**Figure 2:**
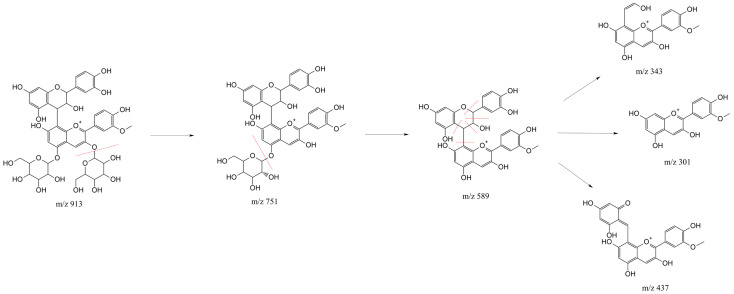
Proposed fragmentation pathway of catechin-(4,8)-peonidin-3,5-diglucoside in positive ion mode.

The proposed structures of fragment ions of catechin-(4,8)-peonidin-3,5-diglucoside are shown in Figure 2. The initial fragmentation event involved the sequential loss of two hexose moieties (162 u each), resulting in the fragment ion at *m*/*z* 751 and the aglycone ion at *m*/*z* 589 [36]. Fragments at *m*/*z* 343, 301, and 427 are derived from the catechin moiety, as previously reported [37]. The ions at *m*/*z* 343 and *m*/*z* 427 can be attributed to fragments resulting from a retro Diels-Alder fragmentation specific to the catechin unit under the high-energy collision conditions. Furthermore, fragment ion at *m*/*z* 343 have also been reported for epicatechin-(4,8)-peonidin-3-glucoside in purple corn [38]. Finally, the peonidin core confirmation is provided by the ion at *m*/*z* 301, which results from the complete cleavage of the catechin unit, yielding the free peonidin aglycone. A similar fragmentation pattern was observed for catechin-(4,8)-pelargonidin-3,5-diglucoside, involving the same types of cleavage, which further supports the identity of both compounds.

Notably, petunidin sophoroside has not been previously reported in purple corn, to the best of our knowledge. Figure 1b,c shows the MS spectra of this anthocyanin in positive and negative ion mode, respectively. A comprehensive search of the literature [4,34,36,38,39,40,41,42,43,44,45,46,47] confirmed that this specific compound has not been previously documented in this source. Although the petunidin aglycone has been detected [48], its conjugation as a sophoroside (petunidin sophoroside) represents a novel finding for this source. The remaining ten anthocyanins matched known compounds previously documented in purple corn studies, as cited before.

**Figure 3:**
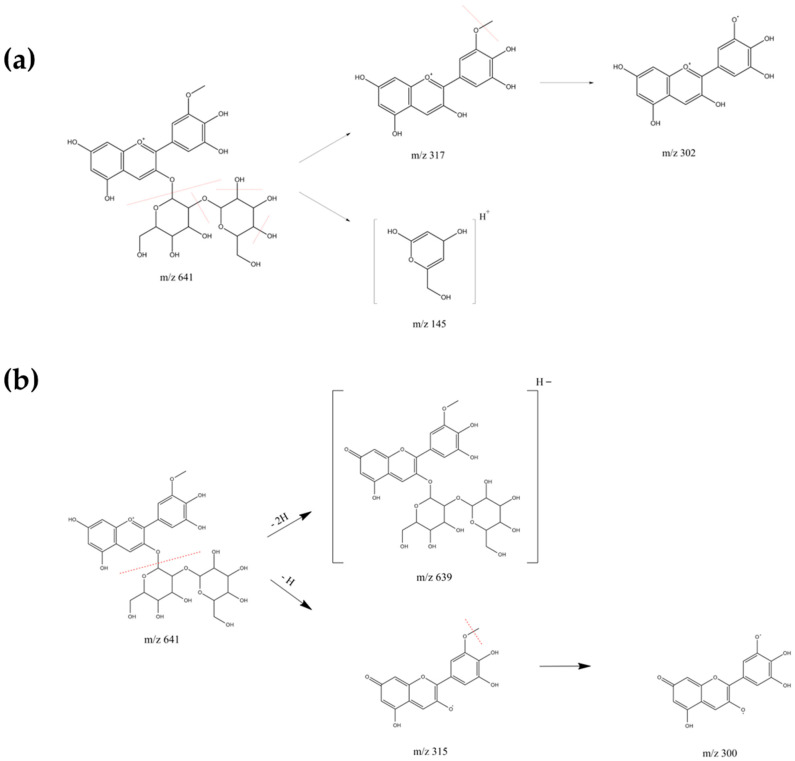
Proposed fragmentation pathway of petunidin sophoroside in positive ion mode (**a**) and negative ion mode (**b**).

The proposed structures of fragment ions (in positive ion mode) of petunidin sophoroside are shown in Figure 3a. The initial fragmentation event involved the loss of 324 u corresponding to the sophoroside moiety, generating an aglycone ion at *m*/*z* 317. A loss of 15 u was observed, corresponding to the cleavage of a –CH_3_ group from the aglycone ion, generating the fragment ion at *m*/*z* 302. Furthermore, while the intact sophoroside fragment (324 u) was not observed, the presence of a characteristic ion at *m*/*z* 145—attributed to specific cleavages within the sophoroside disaccharide moiety—provides supporting evidence for the glycosidic configuration, as documented in previous studies [49]. The proposed structures of the fragment ions of petunidin sophoroside in negative ion mode are shown in Figure 3b. The molecular ion at *m*/*z* 639 originates from the loss of two hydrogen atoms from the starting molecule [50]. The fragment at *m*/*z* 315 corresponds to the loss of 324 u, attributed to the cleavage of the sophoroside moiety. Finally, a further loss of 15 u is observed, corresponding to the cleavage of a –CH_3_ group from the aglycone ion, yielding the fragment ion at *m*/*z* 300. The fragmentation patterns observed in both positive and negative ion modes are consistent with those expected for petunidin sophoroside, thus confirming its molecular identity.

The HPLC-MS analysis revealed a simple anthocyanin composition in the camu-camu peel extract, identifying only two distinct anthocyanins: cyanidin 3-glucoside and delphinidin 3-glucoside. These findings are consistent with previous reports [7,51,52], which, to date, have not identified any additional anthocyanin structures in this botanical source. The retention times and corresponding molecular ions for both compounds are detailed in Table 2.

### 2.2. Anthocyanin Copigmentation

The following coding system was adopted for sample nomenclature throughout this study: The first part of the code indicates the anthocyanin source (PCA for purple corn cob or CCA for camu-camu peel), followed by an underscore and the copigment code (F for ferulic acid, C for cinnamic acid, R for resveratrol, P for polyaspartic acid). A number after the copigment letter indicates the mass ratio (e.g., PCA_F20: PCA with ferulic acid at a 1:20 ratio). For double copigmentation, the codes of the two copigments are combined (e.g., PCA_FP: PCA with ferulic acid and polyaspartic acid at their optimal predetermined ratios, which yield the highest hyperchromic and/or bathochromic effects).

#### 2.2.1. Copigmentation with Phenolic Copigments and Polyaspartic Acid

**Figure 4:**
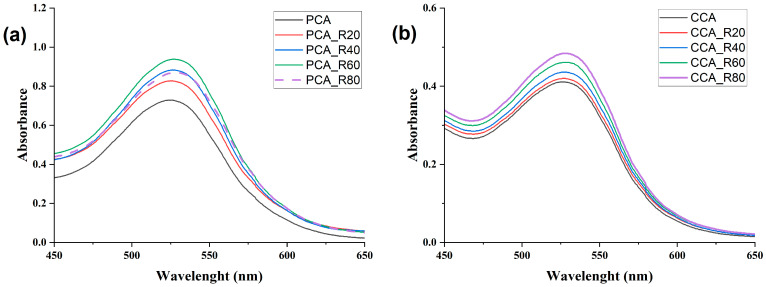
Visible absorption spectra of anthocyanins copigmented with R at different mass ratios: Spectra show the non-copigmented control (PCA or CCA) and samples with increasing resveratrol mass ratios: 1:20 (PCA_R20, CCA_R20), 1:40 (PCA_R40, CCA_R40), 1:60 (PCA_R60, CCA_R60), and 1:80 (PCA_R80, CCA_R80). (**a**) Spectra corresponding to PCA samples. (**b**) Spectra corresponding to CCA samples.

The addition of increasing concentrations of copigments resulted in significant hyperchromic (increased absorbance) and bathochromic (red shift) effects in both PCA and CCA, demonstrating effective stabilization of the flavylium cation (Figure 4, Figures S1 and S2). For PCA copigmented samples, the maximum hyperchromic effect for each copigment was achieved at different optimal ratios (Table 3): PCA_R60 for R, PCA_F80 for F, PCA_C80 for C and PCA_P8 for P. PCA_R60 showed the most pronounced hyperchromic effect (31.37%), indicating that R acts as a superior copigment compared to F and C. However, at higher concentrations (PCA_R80), a notable decrease in maximum absorbance was observed, likely due to the destabilization of the intermolecular stacked anthocyanins [29]. The same behavior was observed in copigmentation with P: PCA_P10 with respect to PCA_P8 and CCA_P18 with respect to CCA_P16. In contrast, PCA_F80 and PCA_C80 exhibited similar hyperchromic effects (18.37% and 20.62%, respectively), suggesting comparable interaction between these phenolic acids and anthocyanins. On the other hand, PCA_C80, PCA_F80, and PCA_R60 showed identical bathochromic shifts (0.45%, 0.51%, and 0.57%, respectively). Notably, copigmentation with P produced the weakest hyperchromic effect, implying that its interactions with PCA (e.g., electrostatic or hydrogen bonding) are less effective than the π-π stacking facilitated by aromatic copigments (F, C and R). Furthermore, PCA_P8 exhibited the weakest bathochromic shift (0.16%) among all copigments.

For CCA copigmentation, the maximum hyperchromic effect for each copigment was achieved at different optimal ratios: CCA_R80 for R, CCA_F80 for F, CCA_C80 for C and CCA_P16 for P. The sample CCA_P16 exhibited the strongest hyperchromic effect (25.09%), demonstrating that P serves as a more effective copigment for CCA compared to R, F, and C. However, CCA_P16 showed the weakest bathochromic shift (0.19%) of all copigments. In contrast, CCA_C80, CCA_F80, and CCA_R80 (1:80 ratios for C, F, and R, respectively) showed comparable hyperchromic enhancements (9.73%, 15.70%, and 15.25%, respectively) and nearly identical bathochromic shifts (0.32%, 0.29%, and 0.32%, respectively).

Previous studies have demonstrated that aspartic acid (the monomeric unit of P) exhibits hyperchromic effects when copigmenting *Fragaria ananassa* anthocyanins [53]. Therefore, it was expected that the polymeric form (P) would similarly induce this hyperchromic effect. Furthermore, computational studies have demonstrated the feasibility of non-covalent interactions between polyaspartate and cyanidin, providing a molecular-level explanation for the observed hyperchromic shifts in copigmented samples [54]. However, minimal bathochromic shifts were observed during P copigmentation of both PCA and CCA, suggesting limited charge transfer interactions between the P polymer and the anthocyanin structures [55,56]. P showed distinct behavior, showing a great hyperchomic shift in CAA, but a modest hyperchromic shift in PCA. This resembles how different polymeric copigments work in different anthocyanin sources. For example, pectin produced a 30% hyperchromic shift with oenin [27] but less than 5% hyperchromic shift with delphinidin-3-glucoside and cyanidin-3-glucoside [57].

The copigments C and F produced similar hyperchromic and bathochromic effects for both PCA and CCA, likely due to their structural similarity as hydroxycinnamic acid derivatives. In contrast, R showed superior hyperchromic enhancement compared to C and F in PCA copigmentation, while exhibiting comparable hyperchromic and bathochromic shifts to hydroxycinnamic acid copigments in CCA stabilization. This differential behavior aligns with previous theoretical studies demonstrating R’s enhanced capacity to form stable non-covalent complexes with cyanidin [12].

The optimally copigmented solutions for subsequent assays were selected based on the highest hyperchromic and/or bathochromic effects. These optimal samples included PCA_P8, PCA_C80, PCA_F80, PCA_R60, CCA_P16, CCA_C80, CCA_F80 and CCA_R80.

#### 2.2.2. Double Copigmentation

The combination of phenolic copigments (F, C or R) with P at their optimal ratios was evaluated for dual copigmentation of anthocyanins (Table 3, Figures S1 and S2). The hyperchromic effect observed for sample PCA_RP (29.54%) was comparable to that of PCA_R60 (31.37%), indicating no synergistic enhancement when combining P with R in the PCA complex. In contrast, sample PCA_CP exhibited significantly greater hyperchromicity (49.24%) than either PCA_C80 (18.37%) or PCA_P8 (7.67%) alone. Similarly, PCA_FP showed markedly increased hyperchromic effects (51.63%) relative to its individual components (PCA_F80: 20.62%; PCA_P8: 7.67%), demonstrating that P potentiates the copigmentation efficacy of both C and F in PCA samples. While PCA_CP and PCA_FP achieved similar hyperchromic enhancements, the greater bathochromic shift in PCA_FP (0.60% vs. 0.38% for PCA_CP) suggests more favorable molecular stacking of F within the ternary complex compared to C. For CCA samples, double copigmentation (CCA_CP, CCA_FP, CCA_RP) resulted in either comparable or reduced hyperchromic effects relative to single phenolic copigments. These doubly copigmented samples also displayed the smallest bathochromic shifts among all tested combinations, implying that ternary complex formation may destabilize the flavylium cation in CCA. This contrasts with previous reports of synergistic stabilization in anthocyanin-phenolic-polymer samples [26], though our findings align with documented cases where phenolic copigments compete with polymers for anthocyanin binding sites [27]. The differential behavior between PCA and CCA samples suggests that anthocyanin structure critically influences ternary complex formation, with P showing preferential enhancement of PCA stabilization while potentially interfering with CCA-copigment interactions. This can be attributed to the differences in the structural complexity of their respective anthocyanin profiles. The PCA extract is characterized by a high diversity of anthocyanins, as evidenced by the identification of twelve distinct compounds. This complex mixture features a wide array of aglycones (cyanidin, peonidin, pelargonidin, and petunidin) and, crucially, varying glycosylation and acylation patterns (including monoglucosides, diglucosides, and both mono- and di-acylated forms with malonic acid). This structural diversity may provide a multitude of potential binding sites and conformational flexibility, allowing for interaction with P. In stark contrast, the CCA profile is straightforward and defined, consisting almost exclusively of cyanidin-3-glucoside and delphinidin-3-glucoside. This aligns with previous studies where the nature of glycosyl moieties has been shown to dictate the stability of anthocyanin-biopolymer complexes, such as those with pectin [57,58].

### 2.3. Thermogravimetric Analysis

**Figure 5:**
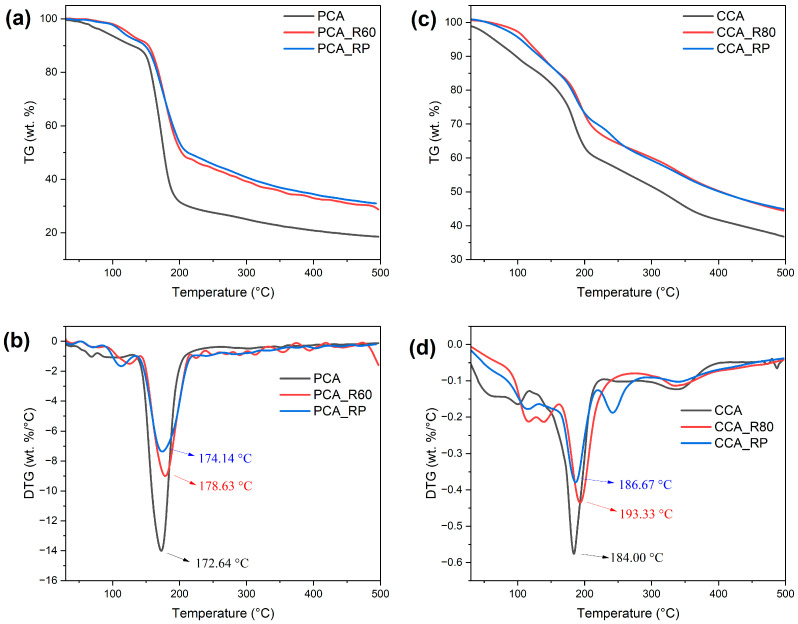
TG and DTG curves of copigmented anthocyanins with resveratrol: (**a**) TG curves of PCA, PCA_R60 and PCA_RP; (**b**) DTG curves of PCA, PCA_R60 and PCA_RP; (**c**) TG curves of CCA, CCA_R80 and CCA_RP; (**d**) DTG curves of CCA, CCA_R80 and CCA_RP.

The thermal degradation profiles of PCA and CCA revealed distinct decomposition patterns through three characteristic stages in their thermogravimetry (TG) and derivative thermogravimetry (DTG) curves (Figure 5, Tables S1 and S2). Both anthocyanin extracts showed an initial mass loss between 30 and 150 °C (PCA: 26.90%; CCA: 13.31%) corresponding to moisture evaporation from the sample surfaces [28]. The primary decomposition phase occurred at 150–200 °C for PCA (54.81% mass loss) and 120–220 °C for CCA (26.92% mass loss), representing thermal degradation of anthocyanin structures through deglycosylation and ring-opening reactions that yield phenolic acids and aldehydes [59]. Notably, CCA exhibited an additional decomposition stage between 300 and 370 °C, absent in PCA, attributed to carbonization of thermal degradation byproducts [60]. DTG analysis yielded Tmax values for the primary decomposition phase of 172.64 °C (PCA) and 184.00 °C (CCA), indicating that CCA has slightly greater inherent thermal stability. The more extensive mass loss observed for PCA during the primary decomposition indicates greater thermal lability and thus weaker thermal stability relative to CCA anthocyanins. These differential thermal behaviors suggest that structural distinctions between the two anthocyanin sources influence decomposition pathways.

Thermogravimetric analysis revealed substantial changes in the degradation patterns of anthocyanins copigmented with R, as seen in their TG and DTG curves (Figure 5, Tables S1 and S2). Both PCA_R60 and CCA_R80 showed reduced moisture loss (1.87% and 2.73%, respectively) compared to their non-copigmented counterparts, indicating enhanced surface hydrophobicity. The PCA_R60 sample exhibited glycosidic bond cleavage between 100 and 140 °C [60], followed by anthocyanin decomposition from 140 to 210 °C with 44.38% mass loss, representing a 10.43% reduction compared to native PCA. Additionally, the Tmax of PCA_R60 increased to 178.63 °C, exceeding that of non-copigmented PCA. Similarly, CCA_R80 exhibited two distinct glycosidic cleavage stages (100–130 °C and 130–155 °C) and anthocyanin decomposition occurring between 155 °C and 220 °C, with only 18.53% mass loss—significantly lower than that of native CCA. Its Tmax rose to 193.33 °C, confirming R’s stabilizing effect. The doubly copigmented samples showed slightly different behavior: PCA_RP maintained similar water loss (2.62%) and decomposition profile (41.61% mass loss at 140–210 °C) to PCA_R60, but with a marginally lower Tmax (174.14 °C). CCA_RP displayed comparable mass loss (20.67% at 130–220 °C) to CCA_R80 but with reduced Tmax (186.67 °C), suggesting that P slightly counteracts R’s stabilization in ternary complexes. Notably, CCA_R80 exhibited an additional decomposition event between 220 °C and 260 °C, potentially indicating the emergence of new degradation pathways in R-CCA complexes. Residual mass analysis confirmed that R-copigmented samples consistently retained higher thermal stability compared to non-copigmented anthocyanins, with double copigmentation exhibiting similar stability to R-only copigmentation. Thermogravimetric analysis of PCA and CCA copigmented with P, C, and F is available in the Supplementary Materials (Figures S3–S5, Tables S1 and S2).

As anticipated, non-copigmented PCA and CCA samples exhibited rapid thermal degradation due to destabilization of the flavylium cation. In all copigmented samples, reduced water loss was observed, suggesting the formation of hydrophobic interactions between anthocyanins and copigments that limit surface moisture adsorption. P demonstrated source-dependent stabilization: while significantly improving PCA thermal resistance, its effect on CCA was less pronounced. This is consistent with reported mechanisms whereby polymers physically encapsulate anthocyanins [27,59,61], suggesting that P may function dually as both copigment and encapsulating agent. 

F showed moderate stabilization of PCA but significantly enhanced CCA thermal resistance, while C paradoxically accelerated PCA degradation while protecting CCA. The observed differential effects arise because stabilization efficacy is governed by specific molecular interactions between the copigment’s structural characteristics and the glycosylation patterns of the anthocyanins. On the other hand, the double copigmentation demonstrated non-significant protective effects compared to single copigment formulations in all samples. Among phenolic copigments, R demonstrated superior protective effects for both PCA and CCA.

### 2.4. FTIR Analysis

**Figure 6:**
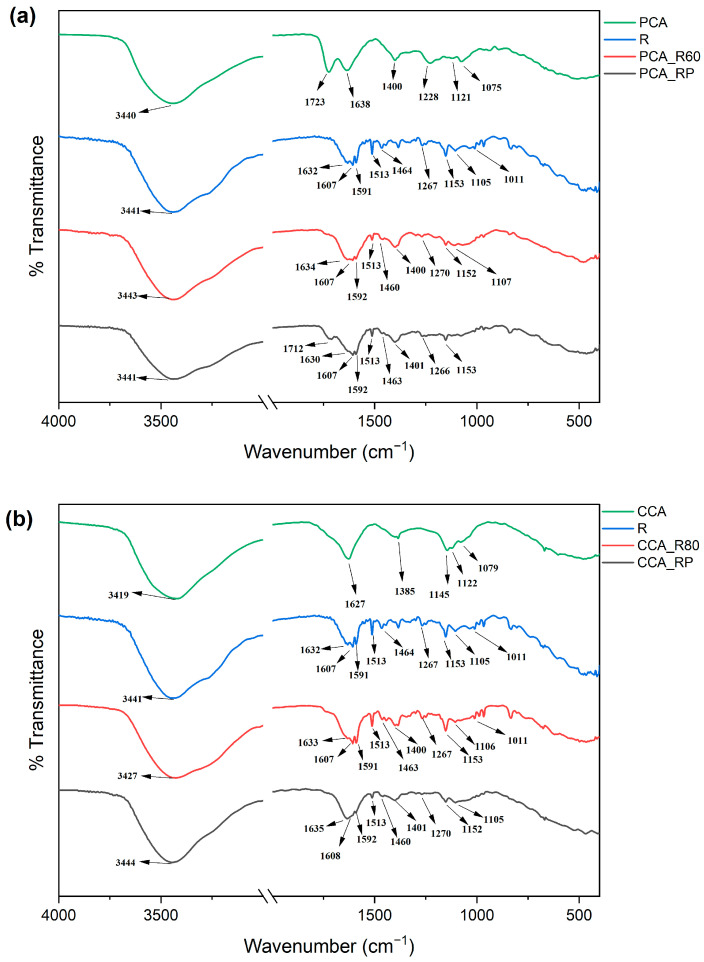
FTIR spectra of copigmented PCA and CCA with resveratrol: (**a**) PCA, R, PCA_R60 and PCA_RP; (**b**) CCA, R, CCA_R80 and CCA_RP.

The formation of hydrogen bonds and π-π interactions between anthocyanins and copigments can be identified through characteristic changes in the wavenumber and/or intensity of the functional groups involved in these non-covalent interactions (e.g., -OH, C-OH, C=O, C=C, etc.). These spectral modifications serve as evidence of non-covalent complex formation between anthocyanins and copigments [26,31]. The FTIR spectra of PCA and CCA revealed characteristic vibrational signatures of their molecular structures (Figure 6). The PCA spectrum exhibited key absorption bands at 3340 cm^−1^ (O-H stretching vibrations), 1723 cm^−1^ (C=O stretching indicative of the quinoidal base form), 1638 cm^−1^ (aromatic C=C stretching), 1400 cm^−1^ (C-OH bending of aromatic hydroxyl groups), and between 1228 and 1075 cm^−1^ (C-O-C stretching of aromatic rings) [62]. The presence of the C=O band at 1723 cm^−1^ confirms the existence of the quinoidal form in the PCA extract [63]. Similarly, the CCA spectrum showed characteristic peaks at 3419 cm^−1^ (O-H stretching), 1627 cm^−1^ (aromatic C=C stretching), 1385 cm^−1^ (aromatic C-OH bending), and in the 1146–1080 cm^−1^ range (C-O-C stretching of aromatic rings). The P spectrum (Figure 6) displayed characteristic absorption bands at 3419 cm^−1^ (O-H stretching vibrations), 1635 cm^−1^ and 1625 cm^−1^ (amide C=O stretching), 1385 cm^−1^ (O-H bending), 1147 cm^−1^ (C-O stretching), and 1119 cm^−1^ (C-N stretching) [64]. The FTIR spectrum of R (Figure 6) exhibited characteristic absorption bands at 3441 cm^−1^ (O-H stretching), 1632, 1607, 1591, 1513, and 1464 cm^−1^ (aromatic C=C stretching), 1267 cm^−1^ (C-OH bending), and 1267, 1153, 1105, and 1011 cm^−1^ (C-O stretching) [65]. Significant spectral modifications were observed in copigmented samples (Figure 6): the CCA_R80 sample showed a shifted O-H stretching band (3427 cm^−1^) compared to pure R, while the C=O band of PCA at 1725 cm^−1^ disappeared in PCAR60, indicating preferential stabilization of the flavylium cation over the quinonoid form when complexed with R. The disappearance of R’s characteristic C-O stretch at 1011 cm^−1^ and intensity reduction of the 1513 cm^−1^ C=C band in PCA_R60, along with the emergence of a new C=C band near 1400 cm^−1^ in both PCA_R60 and CCA_R80, provide evidence of hydrogen bonding and π-π interactions between anthocyanins and R. The introduction of P in doubly copigmented samples further modified these interactions: PCA_RP showed an upshifted C=O band (1712 cm^−1^), suggesting increased quinonoid form preference, while CCA_RP displayed an O-H band shift to 3444 cm^−1^. Key vibrational modes underwent significant changes. The C-OH bending in PCA_RP shifted to 1266 cm^−1^, the C-O stretch at 1107 cm^−1^ disappeared in PCA_RP, and the 1011 cm^−1^ C-O band vanished in CCA_RP. Notably, the C=C bands in PCA_RP (1630 cm^−1^) and CCA_RP (1608 and 1592 cm^−1^) reduced their intensity, confirming altered molecular interactions. These collective spectral changes demonstrate that P addition modifies the original hydrogen bonding and hydrophobic interactions between anthocyanins and R, likely through competitive binding and structural reorganization of the ternary complexes [27]. FTIR analysis of PCA and CCA copigmented with P, C and F can be found in Supplementary Materials [66,67,68] (Figures S6–S8).

The spectroscopic data from both UV-Vis and FTIR analyses provide evidence for the formation of non-covalent complexes between anthocyanins and the various copigments, with the specific interaction mechanisms being highly dependent on the chemical structure of the copigment. For the phenolic copigments—C, F, and R—the observed bathochromic shifts (Δλ) in the UV-Vis spectra are a classic indicator of π–π stacking and charge transfer between the copigment’s conjugated system and the flavylium cation of the anthocyanins, as evidenced by computational methods [12,55,56]. This interaction is further supported by FTIR data, which showed shifts and intensity changes in the aromatic C=C stretching regions. The better stabilizing effect of R, evident in both hyperchromic shifts and thermal stability assays, can be attributed to its unique structural compatibility and superior capacity for intermolecular interactions with the anthocyanin flavylium cation. Unlike the smaller phenolic acids, R features a conjugated system comprising two phenolic rings linked by an ethylene bridge, closely mimicking the core structure of the anthocyanin aglycone itself. This molecular mimicry facilitates an optimal face-to-face π-π stacking interaction, maximizing the overlap between the conjugated samples of both molecules [17]. Concurrently, the significant shifts and broadening of the O-H stretching bands in the FTIR spectra of all copigmented samples, along with changes in the C=O and C-O regions, provide strong evidence for extensive hydrogen bonding. These interactions likely occur between the hydroxyl groups of the anthocyanins and copigments, as well as with the sugar moieties of the glycosylated anthocyanins [69,70]. R possesses three hydroxyl groups, compared to the single hydroxyl in cinnamic acid or the hydroxyl and methoxyl in ferulic acid. This provides a greater number of sites for the formation of strong hydrogen bonds with the hydroxyl and glycosyl groups of the anthocyanins, as well as with surrounding water molecules, forming a more stable complex configuration. At the copigmentation pH of 3.0, P’s backbone is predominantly protonated (pKa ≈ 4.5–4.75) [71], with an estimated degree of deprotonation below 4%. This indicates that the primary mechanism of stabilization at this pH is not electrostatic attraction but rather the formation of an extensive network of hydrogen bonds between the protonated carboxyl groups (-COOH) of P and the hydroxyl and glycosyl moieties of the anthocyanins. The reduction in moisture loss observed in TGA for these samples also suggests that P creates a hydrophobic microenvironment, further shielding the anthocyanin. Finally, in the double copigmentation samples (e.g., PCA_FP, CCA_RP), the spectral changes were not merely additive. The modifications in the O-H and C=O regions compared to the single copigment samples suggest a reorganization of the hydrogen-bonding network and potential cooperative effects, where P may interact with the phenolic copigment itself or create a ternary complex that alters the original interaction geometry.

### 2.5. Copigmented Anthocyanin Stability at pH 7.4

The stability of copigmented PCA and CCA under physiological conditions (pH 7.4, 37 °C) was evaluated by measuring the remaining anthocyanin content after 24 h of incubation, expressed as a percentage of the initial concentration (Equation (1)). This analysis revealed significant protective effects of copigmentation (Table 4). Non-copigmented controls showed the highest susceptibility to degradation, with only 24.26% (PCA) and 45.61% (CCA) of anthocyanins remaining after treatment. This aligns with established evidence that while glycosylated anthocyanins demonstrate moderate stability at physiological pH, their aglycone forms (anthocyanidins) degrade completely within 60 min under these conditions [72]. Phenolic copigments (F, C and R) provided substantial protection through flavylium cation stabilization via hydrophobic interactions and hydrogen bonding. The deprotonated state of F and C at physiological pH likely enhanced complex formation through electrostatic attractions [56]. At physiological pH, R remains predominantly protonated due to its relatively high pKa (>8.0) [73], distinguishing its stabilization mechanism from that of hydroxycinnamic acid copigments (F and C), which deprotonate under these conditions. This protonation state enables R to interact with anthocyanins primarily via π-π stacking and hydrogen bonding, while electrostatic interactions are not favored. These interactions are functionally comparable to those of hydroxycinnamic acids, reflected in their anthocyanin protection after treatment. On the other hand, P alone showed intermediate protection, with PCA_P8 retaining 30.17% anthocyanins (significantly better than control but inferior to phenolic copigments), while CCA_P16 performed remarkably well (51.55% retention), matching phenolic copigment efficacy. At pH 7.4, P exists in its negatively charged polyaspartate form, enabling potential electrostatic interactions with the flavylium cations of PCA and CCA anthocyanins, preventing nucleophilic water attack to the anthocyanin core [74]. The formation of polyelectrolyte complexes between anionic and cationic biopolymers represents a promising advanced strategy for anthocyanin stabilization. While ionic biopolymers like pectin are established anionic copigments [29], their combination with cationic partners such as chitosan in polyelectrolyte complexes has demonstrated superior encapsulation and protective effects of anthocyanins compared to single polymers [31]. Given that P complexes with chitosan have already been explored for other applications [64], the encapsulation of anthocyanins within a P-chitosan polyelectrolyte complexes matrix emerges as a highly promising direction for future research.

**Figure 7:**
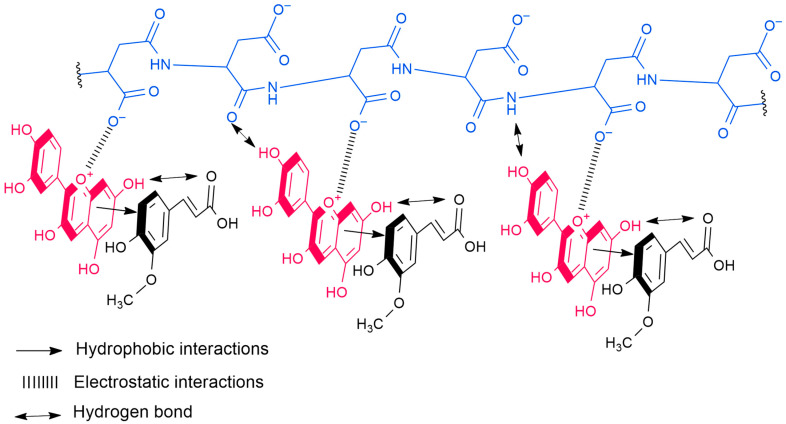
Proposed ternary complex between cyanidin, ferulic acid and polyaspartate, highlighting the interaction between them.

The double copigmentation demonstrated significantly enhanced protective effects compared to single copigment formulations in PCA samples. The PCA_CP system retained 46.02% of anthocyanins after degradation treatment, outperforming PCA_C80 and PCA_P8. Similarly, the PCA_FP combination showed even greater stabilization with 48.31% anthocyanin retention, surpassing all corresponding single-component samples. These results clearly demonstrate that P acts synergistically with phenolic copigments (particularly F and C) to enhance anthocyanin stability in PCA samples. The observed synergistic effect in double copigmentation samples likely arises through a sequential stabilization mechanism. Initially, the phenolic copigments (F and C) stabilize the flavylium cation through π-π stacking and hydrogen bonding interactions, as evidenced by FTIR spectral shifts. This primary stabilization maintains a higher population of positively charged flavylium cations at physiological pH than would exist in uncopigmented or P copigmented samples. The stabilized flavylium species then becomes available for enhanced electrostatic interactions with the carboxylate groups of P, creating a more stable ternary complex at physiological pH (Figure 7).

Consistent with prior analytical observations, no synergistic stabilization was detected in the anthocyanin retention percentages for samples PCA_RP, CCA_CP, CCA_FP and CCA_RP. While R alone showed improved protection of PCA and CCA, its combination with P failed to enhance stability beyond single-copigment performance. This suggests competitive binding between R and P for similar interaction sites on the anthocyanin structure, particularly given R’s superior π-stacking capacity that may displace P’s electrostatic interactions.

### 2.6. In Vitro Oxidative Stress Assessment by TBARS Method

The TBARS assay results demonstrated significant neuroprotective effects for both PCA and CCA against oxidative damage in brain tissue models (Table 5). All copigmented samples showed markedly reduced TBARS levels compared to the oxidative stress-induced control (SI), confirming their antioxidant capacity. The PCA extract alone exhibited substantial protection (120.56 nmol/g TBARS), while the R-copigmented sample (PCA_R60) showed exceptional performance with the lowest observed TBARS value (48.00 nmol/g). Similarly, the CCA extract demonstrated strong inherent antioxidant activity (99.50 nmol/g), with several copigmented formulations—particularly CCA_F80 (60.61 nmol/g), CCA_R80 (44.43 nmol/g), and the double-copigmented combinations CCA_CP (67.82 nmol/g) and CCA_RP (40.36 nmol/g)—outperforming the control (non-stress induced). Notably, R-copigmented samples consistently showed the strongest inhibition of oxidative stress, aligning with previous reports of its neuroprotective efficacy in rat brain models [11,75].

Despite these protective effects, no synergistic interactions between anthocyanins and copigments were observed in either PCA or CCA samples. This absence of synergy may stem from the occupation of critical hydrogen bonding sites during complex formation, rendering these groups unavailable for radical scavenging activities [76]. In some cases, combinations even showed reduced efficacy—for example, CCA_P16 (97.68 nmol/g) and PCA_RP (73.28 nmol/g)—possibly due to pro-oxidant effects at high antioxidant concentrations [77]. Importantly, while copigmentation did not enhance antioxidant synergy, most copigmented samples either matched or exceeded the protection level of the non-induced stress brain control, indicating that the process successfully yields stabilized anthocyanin formulations with preserved—and in some cases enhanced—neuroprotective capacity.

### 2.7. Potential Neurobiological Implications in Alzheimer’s Disease

Beyond the demonstrated protective effect against oxidative stress, the enhanced stability of these anthocyanin-copigment complexes suggests promising broader implications for AD’s therapeutics. The primary pathophysiological hallmarks of AD include the accumulation of amyloid-β (Aβ) plaques and neurofibrillary tangles of hyperphosphorylated tau protein [78,79]. Notably, a growing body of evidence indicates that anthocyanins and their metabolites can interact with these key targets. They have been shown to inhibit Aβ aggregation and fibrillization in vivo [80]. Phenolic copigments used in this study, particularly resveratrol and ferulic acid, are well-documented for their anti-neuroinflammatory properties, inhibition of Aβ formation and reduction of fibrilization [20,81,82,83]. Furthermore, a study has demonstrated that combinations of anthocyanins and phenolic compounds can act as potent and synergistic inhibitors of the enzyme acetylcholinesterase (AChE) [33]. This is critical, as AChE inhibition is a primary strategy to boost cholinergic neurotransmission, which is severely compromised in AD [84].

Therefore, the copigmentation strategy presented here may create a more robust delivery system for these multi-functional neuroprotective compounds. By significantly improving the bioavailability and half-life of the anthocyanins under physiological conditions, we increase the likelihood that sufficient concentrations can reach the brain to concurrently modulate oxidative stress, protein aggregation, AChE activity and neuroinflammation—a multi-targeted approach that is increasingly recognized as essential for effective AD intervention [85]. Future studies should focus on validating these effects in in vitro or in vivo models.

## 3. Materials and Methods

### 3.1. Materials

Purple maize samples were collected in the Ayacucho region of Peru (geographical coordinates: 13°11′29.3″ S, 74°08′54.1″ W). Camu-camu fruit samples were collected in the Loreto region of Peru (geographical coordinates: 5°49′47.7″ S, 76°07′10.7″ W).

### 3.2. Extraction and Purification of Anthocyanins from Purple Corn Cob and Camu-Camu Peel

Purple corn cobs were washed and cut into approximately 5 cm × 5 cm cubes, then dried in an oven at 40 °C for 2 days until constant weight was achieved [86]. The dried purple corn cobs were ground and sieved through a N° 35 mesh. Anthocyanins were extracted using a 1:10 (*w*/*v*) ethanol/0.01% HCl solution at a 1:10 (*w*/*v*) ratio for 30 min, and then concentrated under vacuum. Ripe camu-camu fruits were washed and peeled. The peels were freeze-dried, ground, and sieved through a 35-mesh sieve [51]. Anthocyanins from camu-camu were extracted using ethanol at a 1:15 (*w*/*v*) ratio for 20 min and then concentrated under vacuum. The concentration of monomeric anthocyanins in the extracts was determined using the pH differential method [87]. The dried extracts were redissolved in distilled water (pH 2.0) and centrifuged. The supernatant was loaded onto a Lewatit S 7968 resin column to remove water-soluble impurities. Before use, the resin was pre-washed with distilled water (pH 2.0). The anthocyanin fraction was eluted with ethanol, and the solvent was removed by vacuum evaporation [88].

### 3.3. HPLC-MS Characterization

Three milligrams of each purified anthocyanin sample were dissolved in 3 mL of a methanolic solution (5% acetic acid) and water (5% acetic acid) in a 4:1 ratio, followed by sonication for 5 min. The solution was then filtered through a 0.25-µm disk filter into an HPLC vial. Chromatographic separation was performed using a Dionex Ultimate 3000 UHPLC system (Thermo Scientific, Waltham, MA, USA) with a Luna^®^ Omega C18 column (150 × 2.1 mm, 1.6 µm) maintained at 40 °C. The mobile phase consisted of 0.1% formic acid in water (solvent A) and 0.1% formic acid in acetonitrile (solvent B), delivered at a flow rate of 0.25 mL/min with the following gradient program: initial isocratic hold at 90% A for 1 min, linear gradient to 5% A over 22 min (1–23 min), isocratic hold at 5% A for 2 min (23–25 min), and return to 90% A over 1 min (25–26 min), followed by column re-equilibration for 6 min (26–32 min). Mass spectrometric detection was performed using a Q Exactive Plus hybrid quadrupole-Orbitrap mass spectrometer (Thermo Scientific) operating in positive/negative mode with a spray voltage of 2.8 kV. The ion source parameters included a capillary temperature of 280 °C, sheath gas flow rate of 40 (N_2_), auxiliary gas flow rate of 10 (N_2_), and heater temperature of 300 °C. Full MS scans were acquired over a mass range of 200–1500 *m*/*z* at 70,000 Resolution with an AGC target of 3 × 10^6^ and maximum injection time of 100 ms. Data-dependent MS^2^ scans were performed at 17,500 Resolution with an AGC target of 1 × 10^5^, maximum injection time of 50 ms, and normalized collision energies of 20, 30, and 40 eV.

### 3.4. Anthocyanin Copigmentation

For the preparation of copigmented PCA and CCA solutions, a previously reported methodology was followed with modifications [16]. The phenolic copigments were F, C, and R dissolved in ethanol, while P was dissolved in a pH 3.0 buffer. Equal volumes of PCA or CCA were combined with phenolic copigment solutions at different concentrations to obtain copigmented anthocyanin solutions at mass ratios of 1:20, 1:40, 1:60, and 1:80, which were then diluted with pH 3.0 buffer (e.g., PCA_F20 and CCA_F20 represent PCA-F and CCA-F at a 1:20 mass ratio, respectively). For copigmentation with P, equal volumes of PCA or CCA were mixed with P solutions at varying concentrations to achieve copigmented anthocyanin solutions at mass ratios ranging from 1:2 to 1:18, which were then diluted with ethanol (e.g., PCA_P10 and CCA_P10 represent PCA-P and CCA-P at a 1:10 mass ratio, respectively). For double copigmentation, equal volumes of PCA or CCA, phenolic copigments, and P were combined at the concentrations that yielded the highest hyperchromic and/or bathochromic effects, producing doubly copigmented anthocyanin solutions (e.g., PCA_FP and CCA_FP correspond to the optimal PCA-F-P and CCA-F-P mass ratios, respectively). All mixtures were kept in the dark at room temperature for 20 min to achieve equilibrium. Copigmented solutions were analyzed by UV-Vis spectrophotometry between 450 nm and 650 nm. All samples were compared against control solutions of PCA or CCA without copigments. The effects of copigmentation were evaluated by calculating the hyperchromic and bathochromic shifts [18].

### 3.5. Thermogravimetric Analysis (TG) and Its Derivative (DTG)

The thermal stability of the freeze-dried samples of co-pigmented PCA and CCA was evaluated using a high-resolution thermogravimetric analyzer (TG) (TGA 5500, TA Instruments, New Castle, DE, USA) with an autosampler and equipped with a high-purity nitrogen generator (Peak Scientific Instruments Ltd., Inchinnan, Scotland, UK; model NG3000A-220-EU). A sample of approximately 5 mg was placed in a platinum crucible and heated from 30 to 500 °C at a rate of 10 °C/min. The thermal events of the thermogravimetric curves (TG) and their derivatives (DTG) were analyzed using TRIOS™ software (version 5.4, TA Instruments) to obtain thermal parameters such as the onset temperature of decomposition, the peak degradation temperature, and the decomposed mass in each event until residue. All analyses were performed in triplicate at the Food Processing Laboratory of the Universidad Nacional Intercultural de Quillabamba (UNIQ), Quillabamba, Peru.

### 3.6. Fourier-Transform Infrared Spectroscopy (FTIR) Analysis

Freeze-dried copigmented PCA and CCA samples were homogenized with KBr and analyzed using a Nicolet iS10 FTIR spectrometer. Spectra were acquired in the range of 400 to 4000 cm^−1^ with a resolution of 4 cm^−1^, averaging 32 scans per sample to ensure spectral clarity [16].

### 3.7. Experimental Determination of Copigmented Anthocyanin Stability at pH 7.4

The solutions that demonstrated the highest hyperchromic and/or bathochromic effects were selected as the optimally copigmented solutions. These optimal solutions and double copigmentation samples were diluted with an equal volume of phosphate-buffered saline (PBS, pH 7.4) and adjusted as necessary to maintain consistent experimental conditions [29]. The samples were subsequently incubated in darkness at 37 °C under constant agitation to simulate a physiological environment. The available anthocyanin content was quantified at initial (0 h) and final (24 h) time points using the pH differential method. Remaining anthocyanins were calculated as follows:

(1) $$Remaining\;anthocyanins\;(\%)=\frac{Anthocyanin\;content\;after\;24\;h\;(\frac{mg}{L})}{Anthocyanin\;content\;at\;0\;h\;(\frac{mg}{L})}\times 100$$

### 3.8. TBARS Assay for In Vitro Oxidative Stress Determination

Chicken brains were homogenized in PBS (pH 7.4) at a 10% (*w*/*v*) concentration, followed by centrifugation (5 min, 2500 rpm, 4 °C). Oxidative stress induction was performed according to the literature protocol with modifications [89]. Briefly, 4 mM ascorbic acid (45 μL) and 2 mM FeSO_4_ (15 μL) were mixed with 900 μL brain homogenate and 60 μL sample, followed by 30 min incubation. Subsequently, 300 μL of the reaction mixture was combined with 600 μL 10% trichloroacetic acid and heated at 100 °C for 15 min. Then, 900 μL 0.67% thiobarbituric acid was added, and the mixture was heated again at 100 °C for 30 min. After centrifugation (10 min, 7000 rpm), the absorbance of the supernatant was measured at 535 nm using UV-Vis spectrophotometry. Thiobarbituric acid reactive species (TBARS) concentration was calculated using Equation (1):

(2) $$\frac{nmol\;TBARS}{g\;brain\;tissue}=\frac{Amp.V_{t}}{\varepsilon .l.V_{mp}.C_{hd}}10^{9}$$

where *A_mp_* represents the absorbance of the test sample at 535 nm, *V_t_* is the total reaction volume (in mL), ε is the molar extinction coefficient of the malondialdehyde-thiobarbituric acid [MDA-(TBA)_2_] complex (1.56 × 10^5^ M^−1^cm^−1^), l is the path length (1 cm), and *C_hd_* corresponds to the brain homogenate concentration. This equation allowed for the quantification of lipid peroxidation products generated during the oxidative stress induction process.

### 3.9. Statistical Analysis

The results of the three independent experiments were expressed as the mean ± standard deviation. Data were analyzed by one-way ANOVA followed by Tukey’s test at a 5% level of significance (*p*-value < 0.05).

## 4. Conclusions

A new anthocyanin in purple corn extracts, petunidin sophoroside, was identified by HPLC-MS. A more comprehensive fragmentation analysis is presented, providing strong validation for the structures of catechin-(4,8)-peonidin-3,5-diglucoside and catechin-(4,8)-pelargonidin-3,5-diglucoside. Copigmentation with R showed greater hyperchromic effects than C and F as copigments for PCA. The polymeric copigment P demonstrated a remarkable and distinct copigmentation capacity, acting as a superior copigment for CCA and exhibiting a synergistic hyperchromic effect when combined with phenolic acids (C and F) in PCA samples. These results demonstrate that P functions as a new versatile copigment for anthocyanins. Copigmentation with R significantly improved the thermal and pH stability of both anthocyanin sources, attributed to optimal π-π stacking and hydrogen bonding with the flavylium cation, as confirmed by FTIR and TG analysis. The combination of P with phenolic copigments (C and F) further enhanced the stability of PCA at physiological pH (7.4) through the formation of a more stable ternary complex. Regarding oxidative stress, all copigmented anthocyanin formulations exhibited significant neuroprotective effects in a brain tissue model. Samples copigmented with R consistently provided the strongest protection against lipid peroxidation. The use of the copigmentation process will enable the production of more stable anthocyanin extracts with good protective activity against oxidative stress, which is expected to improve the treatment of AD.

## Figures and Tables

**Table 1:** Major anthocyanins identified in purple corn cob (*Zea mays* L.) extract.

Anthocyanin	Retention Time (min)	Molecular Ion [M]^+^ (*m*/*z*)	MS Ions (*m*/*z*)
Cyanidin-3-malonylglucoside	3.53	535	177, 287
Peonidin-3-glucoside	3.57	463	112, 286, 301, 383
Cyanidin-3-glucoside	3.64	449	167, 244, 287
Catechin-(4,8)-pelargonidin-3,5-diglucoside	3.67	883	271, 313, 407, 559, 721
Pelargonidin-3-glucoside	4.16	433	68, 130, 235, 271, 307
Catechin-(4,8)-peonidin-3,5-diglucoside	4.47	913	301, 343, 437, 589, 751
Cyanidin-3-malonylglucosyl-5-glucoside	4.52	697	177, 287, 449, 535
Pelargonidin-3-malonylglucoside	15.34	519	187, 271, 433, 475
Peonidin-3-malonylglucoside	15.76	549	185, 286, 301, 505
Pelargonidin-3-dimalonylglucoside	16.62	605	137, 177, 271, 425
Peonidin-3-dimalonylglucoside	16.81	635	137, 177, 301, 436
Petunidin sophoroside	17.57	641 639 *	145, 302, 317, 479 300 *, 315 *

* MS data acquired in negative ion mode; all other fragments were obtained in positive ion mode.

**Table 2:** Major anthocyanins identified in camu-camu peel (*Myrciaria dubia*) extract.

Anthocyanin	Retention Time (min)	Molecular Ion [M]^+^ (*m*/*z*)	MS Ions (*m*/*z*)
Cyanidin-3-glucoside	3.50	449	68, 153, 229, 287, 375
Delphinidin-3-glucoside	16.82	465	85, 153, 267, 303

**Table 3:** Hyperchromic and bathochromic effects of copigmented PCA and CCA with P, C, F, and R and double-copigmented samples.

PCA Samples	%A	%λ	CCA Samples	%A	%λ
PCA_P8	7.67 ± 0.26 ^d^	0.16 ± 0.05 ^d^	CCA_P16	25.09 ± 4.37 ^a^	0.19 ± 0.00 ^a^
PCA_C80	18.37 ± 1.79 ^c^	0.45 ± 0.06 ^bc^	CCA_C80	9.73 ± 0.98 ^bcd^	0.32 ± 0.05 ^a^
PCA_CP	49.24 ± 1.49 ^a^	0.38 ± 0.05 ^c^	CCA_CP	7.38 ± 0.51 ^cd^	−1.08 ± 0.15 ^c^
PCA_F80	20.62 ± 0.10 ^c^	0.51 ± 0.00 ^abc^	CCA_F80	15.70 ± 3.05 ^b^	0.29 ± 0.00 ^a^
PCA_FP	51.63 ± 0.64 ^a^	0.60 ± 0.00 ^a^	CCA_FP	5.43 ± 0.61 ^d^	−0.45 ± 0.06 ^b^
PCA_R60	31.38 ± 2.66 ^b^	0.57 ± 0.06 ^ab^	CCA_R80	15.25 ± 3.00 ^b^	0.32 ± 0.05 ^a^
PCA_RP	29.54 ± 1.03 ^b^	0.57 ± 0.10 ^ab^	CCA_RP	12.25 ± 0.57 ^bc^	−0.51 ± 0.22 ^b^

Parameters: %ΔA = Hyperchromic shift; %Δλ = Bathochromic shift. Data were analyzed by one-way ANOVA followed by Tukey’s post hoc test. Different superscript letters (a–d) within the same column indicate significant differences (*p* ≤ 0.05).

**Table 4:** Remaining copigmented PCA and CCA with P, C, F, and R and double-copigmented samples after 24 h at pH 7.4 and 37 °C.

PCA Samples	Remaining Anthocyanins (%)	CCA Samples	Remaining Anthocyanins (%)
PCA	24.26 ± 1.08 ^e^	CCA	45.61 ± 1.56 ^b^
PCA_P8	30.17 ± 0.57 ^d^	CCA_P16	51.55 ± 2.63 ^a^
PCA_C80	38.82 ± 0.88 ^bc^	CCA_C80	54.72 ± 1.08 ^a^
PCA_CP	46.02 ± 0.68 ^a^	CCA_CP	55.61 ± 1.49 ^a^
PCA_F80	40.86 ± 1.39 ^b^	CCA_F80	56.57 ± 2.78 ^a^
PCA_FP	48.31 ± 1.28 ^a^	CCA_FP	56.10 ± 2.19 ^a^
PCA_R60	38.85 ± 1.49 ^bc^	CCA_R80	53.92 ± 1.36 ^a^
PCA_RP	37.16 ± 1.31 ^c^	CCA_RP	55.84 ± 1.02 ^a^

Data were analyzed by one-way ANOVA followed by Tukey’s post hoc test. Different superscript (a–e) letters within the same column indicate significant differences (*p* ≤ 0.05).

**Table 5:** TBARS (nmol/g) values of copigmented PCA and CCA with P, C, F, and R and double-copigmented samples.

Sample	TBARS (nmol/g)	Sample	TBARS (nmol/g)
Control	106.50 ± 7.70 ^bc^	Control	106.50 ± 7.70 ^b^
SI	172.64 ± 14.16 ^a^	SI	172.64 ± 14.16 ^a^
**PCA Samples**		**CCA Samples**	
P	76.12 ± 6.13 ^d^	P	54.27 ± 11.45 ^de^
C	83.77 ± 9.84 ^cd^	C	98.85 ± 3.94 ^b^
F	88.51 ± 2.91 ^cd^	F	77.94 ± 7.76 ^bcd^
R	51.14 ± 6.82 ^ef^	R	44.80 ± 5.14 ^e^
PCA	120.56 ± 7.55 ^b^	CCA	99.50 ± 13.12 ^b^
PCA_P8	100.90 ± 5.44 ^bc^	CCA_P16	97.68 ± 14.14 ^b^
PCA_C80	90.25 ± 7.28 ^cd^	CCA_C80	77.80 ± 7.31 ^bcd^
PCA_CP	105.48 ± 5.91 ^bc^	CCA_CP	67.82 ± 11.24 ^cde^
PCA_F80	115.46 ± 8.73 ^b^	CCA_F80	60.61 ± 10.45 ^cde^
PCA_FP	105.41 ± 9.25 ^bc^	CCA_FP	86.47 ± 8.01 ^bc^
PCA_R60	48.00 ± 3.72 ^f^	CCA_R80	44.43 ± 3.03 ^e^
PCA_RP	73.28 ± 6.78 ^de^	CCA_RP	40.36 ± 1.46 ^e^

Data were analyzed by one-way ANOVA followed by Tukey’s post hoc test. Different superscript letters (a–f) within the same column indicate significant differences (*p* ≤ 0.05).

## Data Availability

Data supporting reported results can be found at https://cybertesis.unmsm.edu.pe/item/d0b7fa8a-234c-4172-a1e2-dd5fb96c4e24 (accessed on 13 November 2025).

## Supplementary Materials

The following supporting information can be downloaded at: https://www.mdpi.com/article/10.3390/molecules30234553/s1. Figure S1: Visible absorption spectra of copigmented CCA: (a) CCA copigmented with C; (b) CCA copigmented with F; (c) CCA copigmented with P; (d) Double-copigmented CCA; Figure S2: Visible absorption spectra of copigmented PCA: (a) PCA copigmented with C; (b) PCA copigmented with F; (c) PCA copigmented with P; (d) Double-copigmented PCA; Figure S3: TG and DTG curves of copigmented anthocyanins with polyaspartic acid: (a) TG curves of PCA and PCA_P8; (b) DTG curves of PCA and PCA_P8; (c) TG curves of CCA and CCA_P16; (d) DTG curves of CCA and CCA_P16; Figure S4: TG and DTG curves of copigmented anthocyanins with cinnamic acid: (a) TG curves of PCA, PCA_C80 and PCA_CP; (b) DTG curves of PCA, PCA_C80 and PCA_CP; (c) TG curves of CCA, CCA_C80 and CCA_CP; (d) DTG curves of CCA, CCA_C80 and CCA_CP; Figure S5: TG and DTG curves of copigmented anthocyanins with ferulic acid: (a) TG curves of PCA, PCA_F80 and PCA_FP; (b) DTG curves of PCA, PCA_F80 and PCA_FP; (c) TG curves of CCA, CCA_F80 and CCA_FP; (d) DTG curves of CCA, CCA_F80 and CCA_FP; Figure S6: FTIR spectra of copigmented anthocyanins with polyaspartic acid: (a) FTIR spectra of PCA, P and PCA_P8; (b) FTIR spectra of CCA, P, CCA_P16 and CCA_P16; Figure S7: FTIR spectra of copigmented anthocyanins with cinnamic acid: (a) FTIR spectra of PCA, C, PCA_C80 and PCA_CP; (b) FTIR spectra of CCA, C, CCA_C80 and CCA_CP; Figure S8: FTIR spectra of copigmented anthocyanins with ferulic acid: (a) FTIR spectra of PCA, F, PCA_F80 and PCA_FP; (b) FTIR spectra of CCA, F, CCA_F80 and CCA_FP; Table S1: Thermal stability data of copigmented PCA; Table S2: Thermal stability data of copigmented CCA.
