# Supplementary material for: Influence of Copigmentation on the Stability and Oxidative Stress of Anthocyanins from Purple Corn and Camu-Camu
Source: Molecules. 2025 Nov 26;30(23):4553. doi: 10.3390/molecules30234553 (PMC12693339; doi:10.3390/molecules30234553)
Supplement: Supplementary file 1 [file molecules-30-04553-s001.zip › molecules-3929583-supplementary.pdf]

Supplementary Materials

1. UV-Vis of Anthocyanins copigmentation
2. Thermogravimetric analysis of copigmented anthocyanins
3. FTIR analysis of copigmented anthocyanins

1. Anthocyanins copigmentation

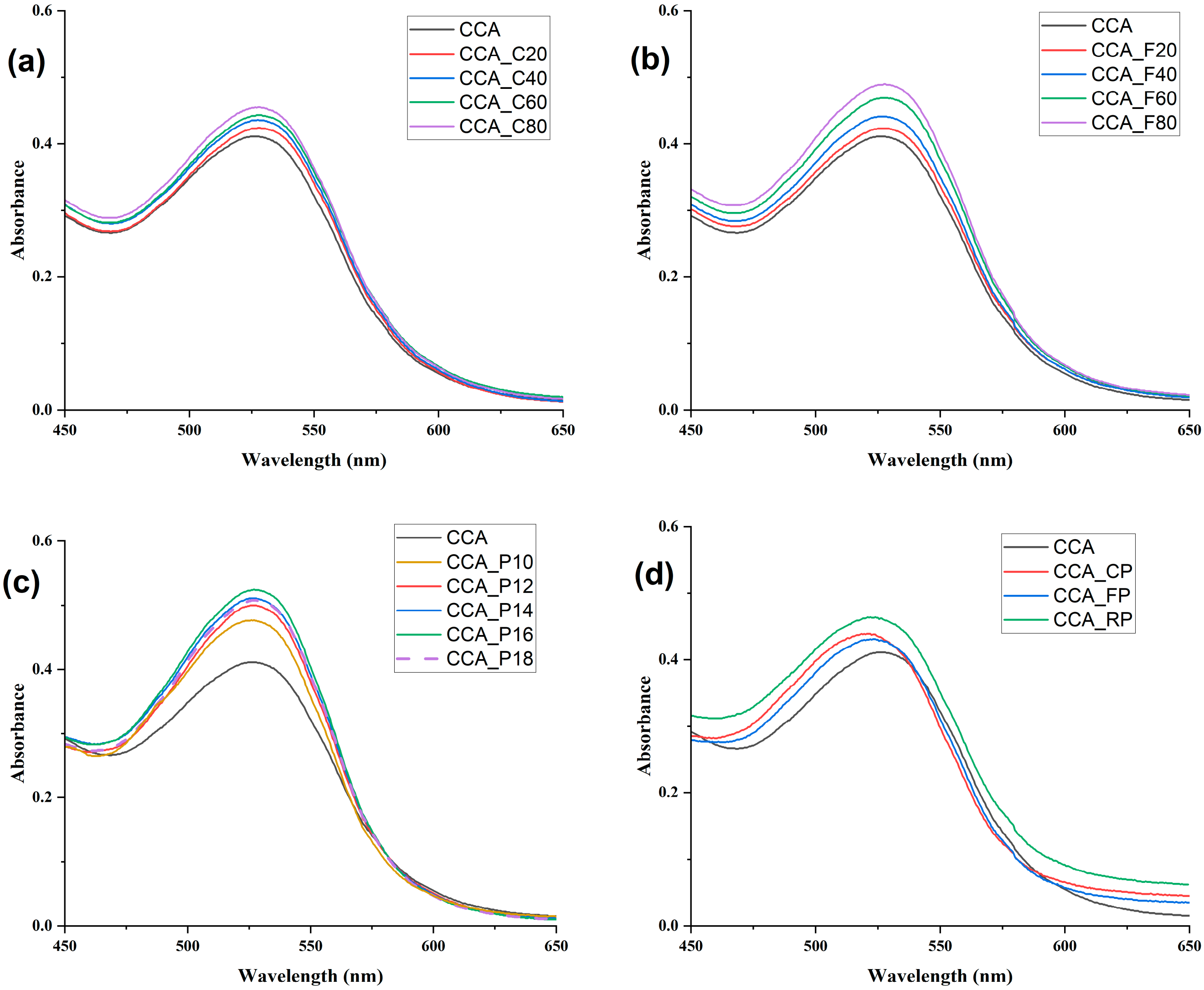

Figure S1. Visible absorption spectra of copigmented CCA: (a) CCA copigmented with C; (b) CCA copigmented with F; (c) CCA copigmented with P; (d) Double copigmented CCA.

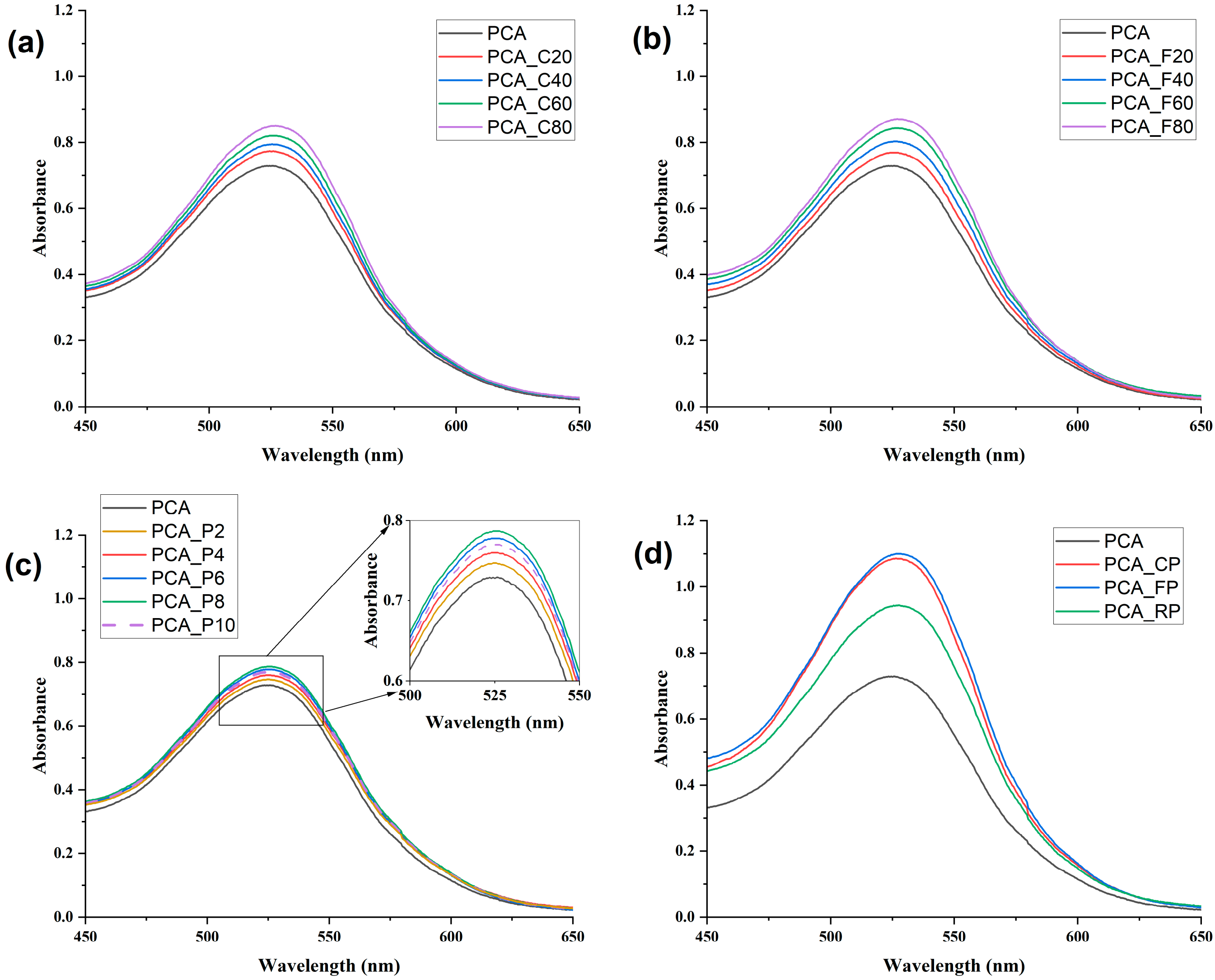

Figure S2. Visible absorption spectra of copigmented PCA: (a) PCA copigmented with C; (b) PCA copigmented with F; (c) PCA copigmented with P; (d) Double copigmented PCA.

2. Thermogravimetric analysis of copigmented anthocyanins

All thermogravimetric data of both sources of anthocyanins can be found in Table S1-2. TG and DTG curves for PCA_P8 and CCA_P16 are presented in Figure S3. Both copigmented samples exhibited reduced moisture loss (PCA_P8: 6.71%; CCA_P16: 10.46%) compared to their non-copigmented counterparts, indicating enhanced surface hydrophobicity due to interactions with P. The anthocyanin decomposition phase occurred between 150–200 °C for PCA_P8 (57.29% mass loss) and 125–220 °C for CCA_P16 (26.76% mass loss), with mass loss magnitudes similar to those observed in non-copigmented anthocyanins. However, the Tmax of PCA_P8 increased to 180.30 °C, reflecting a slower degradation process relative to native PCA, while CCA_P16 exhibited a Tmax of 183.67 °C, comparable to that of CCA. Notably, the onset of anthocyanin degradation in CCA_P16 was delayed to 125 °C versus 120 °C in CCA, suggesting a modest stabilizing effect. Both copigmented samples yielded higher residual mass than non-copigmented anthocyanins, further supporting P's role in promoting thermal stability. The thermal deg-

radation profile observed for the P-anthocyanin complexes resembles the behavior reported for other established encapsulating polymers, such as chitosan or starch, when complexed with anthocyanins [59,61]. This similarity in thermal behavior suggests that P, beyond its role as a copigment, may also be functioning as an encapsulating agent, forming a protective matrix that delays the volatilization and decomposition of the anthocyanins. This dual potential—as both a copigment and an encapsulant—positions P as a versatile polymer for the development of advanced anthocyanin stabilization strategies.

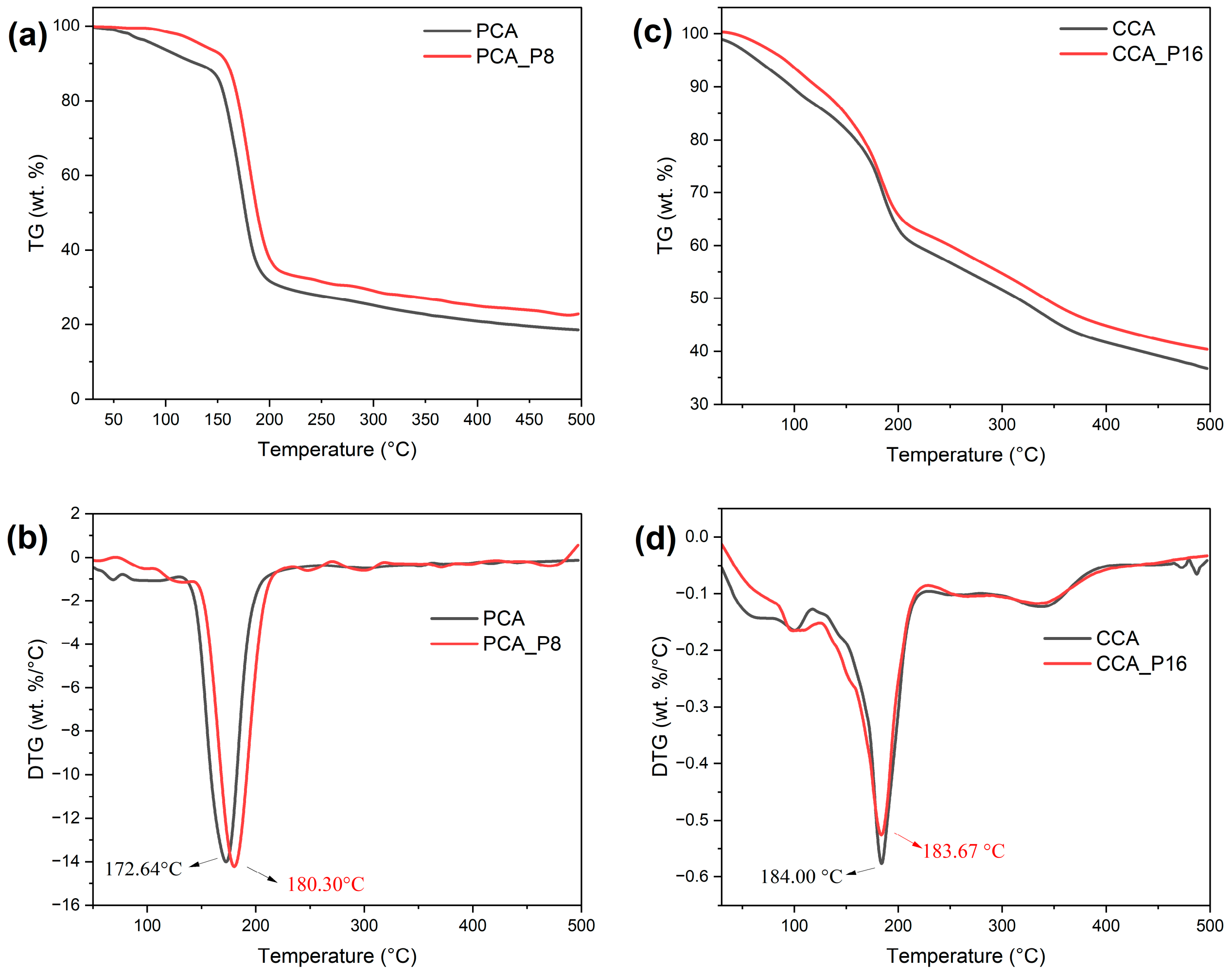

Figure S3. TG and DTG curves of copigmented anthocyanins with polyaspartic acid: (a) TG curves of PCA and PCA_P8; (b) DTG curves of PCA and PCA_P8; (c) TG curves of CCA and CCA_P16; (d) DTG curves of CCA and CCA_P16.

The thermal degradation profiles of anthocyanins copigmented with C, both alone and in combination with P, are shown in Figure S4. Samples PCA_C80 and CCA_C80 exhibited reduced moisture loss (1.9% and 4.44%, respectively) compared to non-copigmented anthocyanins, indicating enhanced surface hydrophobicity. A new decomposition event attributed to glycosidic bond cleavage [60] was observed in both systems, occurring at 110–140 °C for PCA_C80 and 105–170 °C for CCA_C80 (more pronounced in the latter), suggesting that C promotes glycosidic dissociation in both anthocyanin types. PCA_C80 showed anthocyanin decomposition from 140–200 °C (65.54% mass loss), exceeding that of native PCA, while CCA_C80 decomposed from 170–220 °C (17.68% mass

loss), lower than native CCA. The Tmax of PCA_C80 increased to 181.96 °C, indicating slower degradation than PCA, whereas CCA_C80 exhibited a Tmax of 186.34 °C, similar to CCA. Both samples yielded lower residual mass than non-copigmented anthocyanins. The double copigmentation samples (PCA_CP and CCA_CP) behaved similarly to their single copigment counterparts. PCA_CP retained low water loss (1.9%), matching PCA_C80, while CCA_CP showed higher moisture loss (9.71%) than CCA_C80, possibly due to weakened hydrophobic interactions upon P addition. Glycosidic cleavage occurred at 110–140 °C (PCA_CP) and 120–170 °C (CCA_CP). Anthocyanin decomposition ranges and mass losses remained consistent: PCA_CP at 140–200 °C (62.91% mass loss) and CCA_CP at 170–220 °C (15.55% mass loss). Tmax values were nearly identical (PCA_CP: 180.30 °C; CCA_CP: 184.17 °C), and residual masses showed no significant changes. These results demonstrate that adding P to C-anthocyanin copigmentation samples does not substantially alter thermal stability.

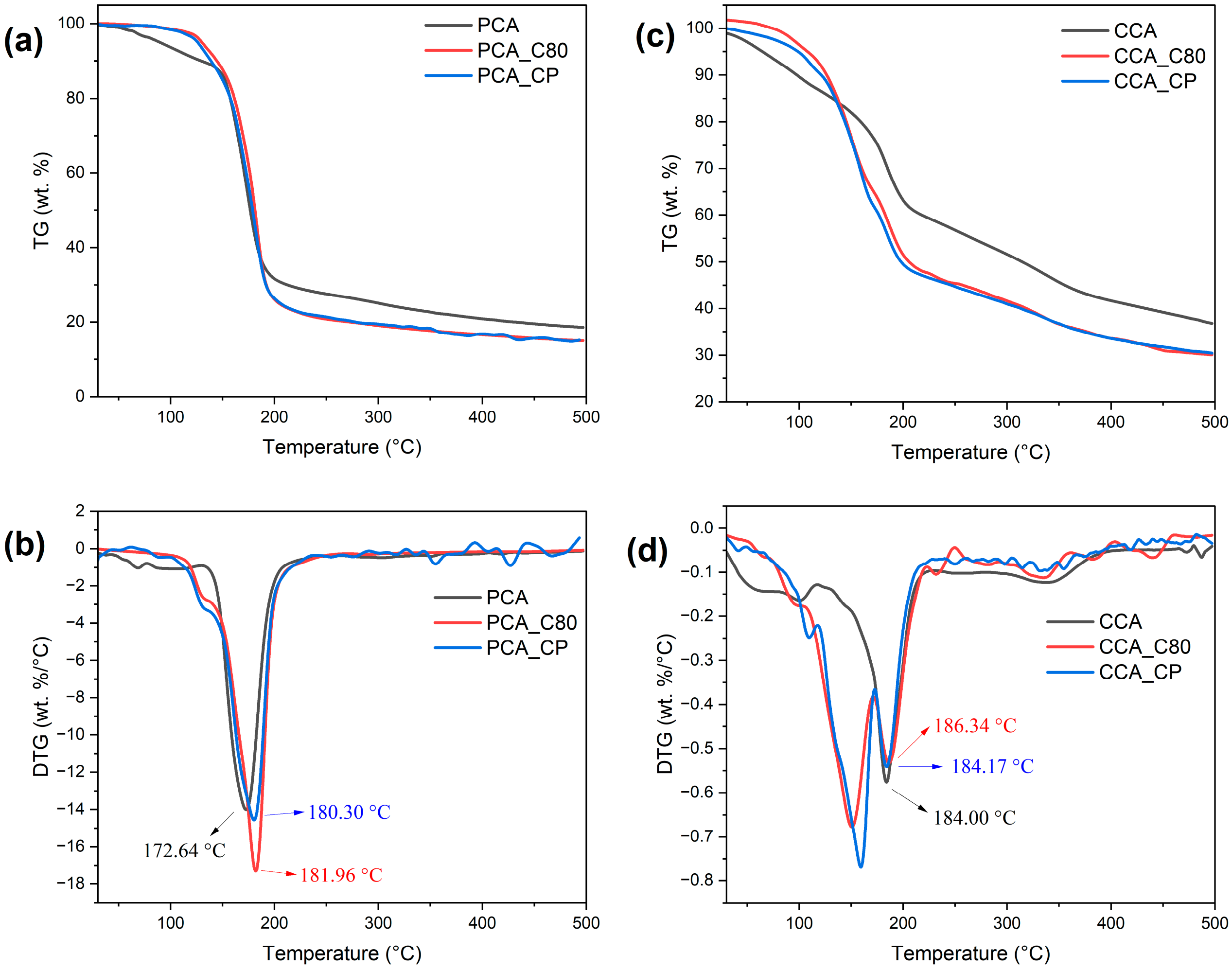

Figure S4. TG and DTG curves of copigmented anthocyanins with cinnamic acid: (a) TG curves of PCA, PCA_C80 and PCA_CP; (b) DTG curves of PCA, PCA_C80 and PCA_CP; (c) TG curves of CCA, CCA_C80 and CCA_CP; (d) DTG curves of CCA, CCA_C80 and CCA_CP.

The thermal degradation profiles of anthocyanins copigmented with F, both alone and in combination with P, are presented in Figure S5. Samples PCA_F80 and CCA_F80 exhibited reduced moisture loss (8.34% and 3.79%, respectively) compared to non-copigmented anthocyanins, indicating enhanced surface hydrophobicity due to F interactions. CCA_F80 displayed a distinct glycosidic bond cleavage event between 90–140°C, while PCA_F80 showed anthocyanin decomposition from 140–200 °C (55.45% mass loss), similar to native PCA. CCA_F80 decomposed from 140–220 °C (29.33% mass loss), comparable to native CCA. The Tmax of PCA_F80 increased to 177.63 °C, indicating slightly slower degradation than PCA, while CCA_F80 exhibited a Tmax of 194.00 °C, confirming slower degradation than CCA. Residual masses for both samples were similar to non-copigmented anthocyanins. Double copigmentation samples (PCA_FP and CCA_FP) showed both similarities and differences relative to single F copigmentation. PCA_FP demonstrated further reduced water loss (2.70%) compared to PCA_F80, indicating strengthened hydrophobic interactions with P addition, while CCA_FP maintained similar moisture loss (3.79%) to CCA_F80. A new glycosidic cleavage event emerged in PCA_FP at 100–150 °C, absent in PCA_F80, suggesting that P promotes glycosidic dissociation in PCA. CCA_FP retained its cleavage event at 90–130 °C, mirroring CCA_F80. Anthocyanin decomposition ranges and mass losses remained consistent: PCA_FP at 150–200 °C (53.04% mass loss) and CCA_FP at 130–210 °C (28.94% mass loss). Notably, PCA_FP's Tmax rose to 181.63 °C, slightly exceeding PCA_F80, indicating P further slows PCA degradation. In contrast, CCA_FP's Tmax (185.67 °C) was similar to CCA_F80, implying P does not significantly influence CCA's thermal stability. Residual masses were unchanged in both double copigmentation samples.

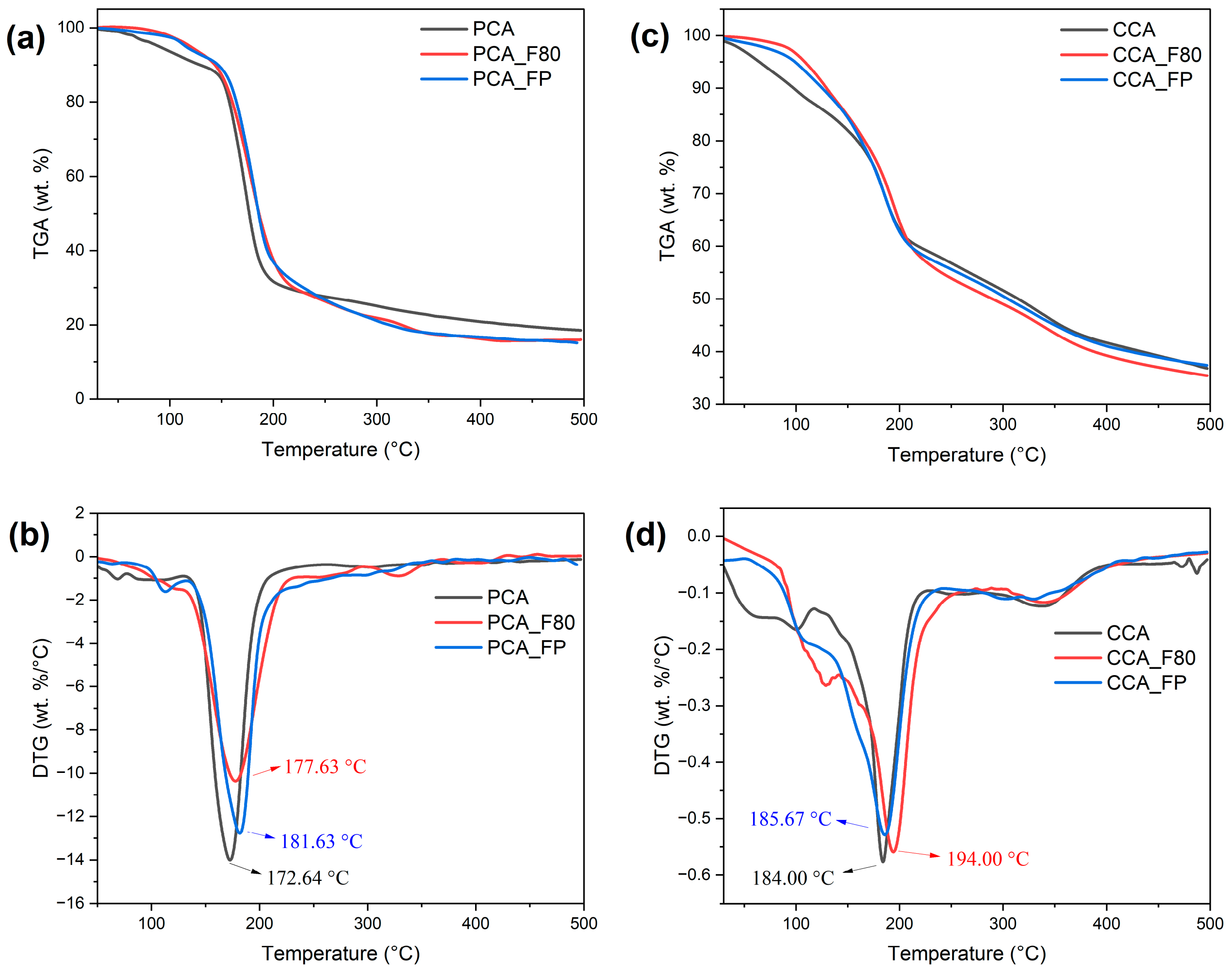

Figure S5. TG and DTG curves of copigmented anthocyanins with ferulic acid: (a) TG curves of PCA, PCA_F80 and PCA_FP; (b) DTG curves of PCA, PCA_F80 and PCA_FP; (c) TG curves of CCA, CCA_F80 and CCA_FP; (d) DTG curves of CCA, CCA_F80 and CCA_FP.

Table S1. Thermal stability data of copigmented PCA.

Sample	Decomposition range (°C)	Mass loss (%)	Tmax (°C)	Remaining mass at 500 °C (%)
PCA	30-150	26.90	-	18.55
	150-200	54.81	172.64	
PCA_P8	30-150	6.71	-	22.77
	150-200	57.29	180.3	
PCA_C80	30-110	1.90	-	14.95
	110-140	7.04	130.23	
	140-200	65.54	181.96	
PCA_CP	30-110	1.90	-	15.12
	110-140	8.66	130.72	
	140-200	62.91	180.3	
PCA_F80	30-140	8.34	-	16.02
	140-200	55.45	177.63	

PCA_FP	30-100	2.70	-	15.12
	100-150	8.06	112.75	
	150-200	53.04	181.63	
PCA_R60	30-100	1.87	-	28.69
	100-140	5.77	124.61	
	140-210	44.38	178.63	
PCA_RP	30-100	2.62	-	30.99
	100-140	4.21	112.9	
	140-210	41.61	174.14	

Table S2. Thermal stability data of copigmented CCA.

Sample	Decomposition range (°C)	Mass loss (%)	Tmax (°C)	Remaining mass at 500 °C (%)
CCA	30-120	13.31	100.28	36.8
	120-220	26.92	184.00	
CCA _P16	30-125	10.46	98.78	40.38
	125-220	26.76	183.67	
CCA _C80	30-105	4.44	92.49	30.06
	105-170	30.01	150.54	
	170-220	17.68	186.34	
CCA _CP	30-120	9.71	109.44	30.42
	120-170	27.77	159.36	
	170-220	15.55	184.17	
CCA _F80	30-90	3.79	-	35.38
	90-140	10.53	128.75	
	140-220	29.33	194.00	
CCA _FP	30-90	3.79	-	37.37
	90-130	7.15	104,09	
	130-210	28.94	185.67	
CCA _R80	30-100	2.73	-	44.92
	100-130	5.91	116.59	
	130-155	5.19	138.90	
	155-220	18.53	193.33	
CCA _RP	30-90	3.05	-	44.42
	90-130	6.45	115.09	
	130-220	20.67	186.67	
	220-260	6.4	241.94	

3. FTIR analysis of copigmented anthocyanins.

The FTIR spectrum of P (Figure S6) exhibited characteristic bands at 3419 cm^{-1} (O–H stretching), 1635 and 1625 cm^{-1} (amide C=O stretching), 1385 cm^{-1} (O–H bending), 1147 cm^{-1} (C–O stretching), and 1119 cm^{-1} (C–N stretching) [64]. For the PCA_P8 sample, the O–H bending band of P at 1385 cm^{-1} decreased in intensity, while the C–OH stretching band of PCA shifted to 1468 cm^{-1}, indicating hydrogen bond formation between PCA and P. The disappearance of the C=O stretching band of PCA suggests preferential stabilization of the flavylium cation over the quinonoid form upon copigmentation with P. New bands emerged at 1595, 1568, and 1468 cm^{-1}, attributed to C=C stretching of flavonoid aromatic rings, likely resulting from enhanced resonance of PCA aromatic rings due to a better stacking state when copigmented with P. In the CCA_P16 sample, the

intensity and wavenumber of C–O–C stretching bands of CCA decreased, and the O–H stretching band shifted to 3444 cm⁻¹. The intensity of the O–H bending band of P at 1385 cm⁻¹ also diminished, confirming hydrogen bond formation between CCA and P. These spectral changes collectively demonstrate that P interacts with both anthocyanin sources mainly through hydrogen bonding.

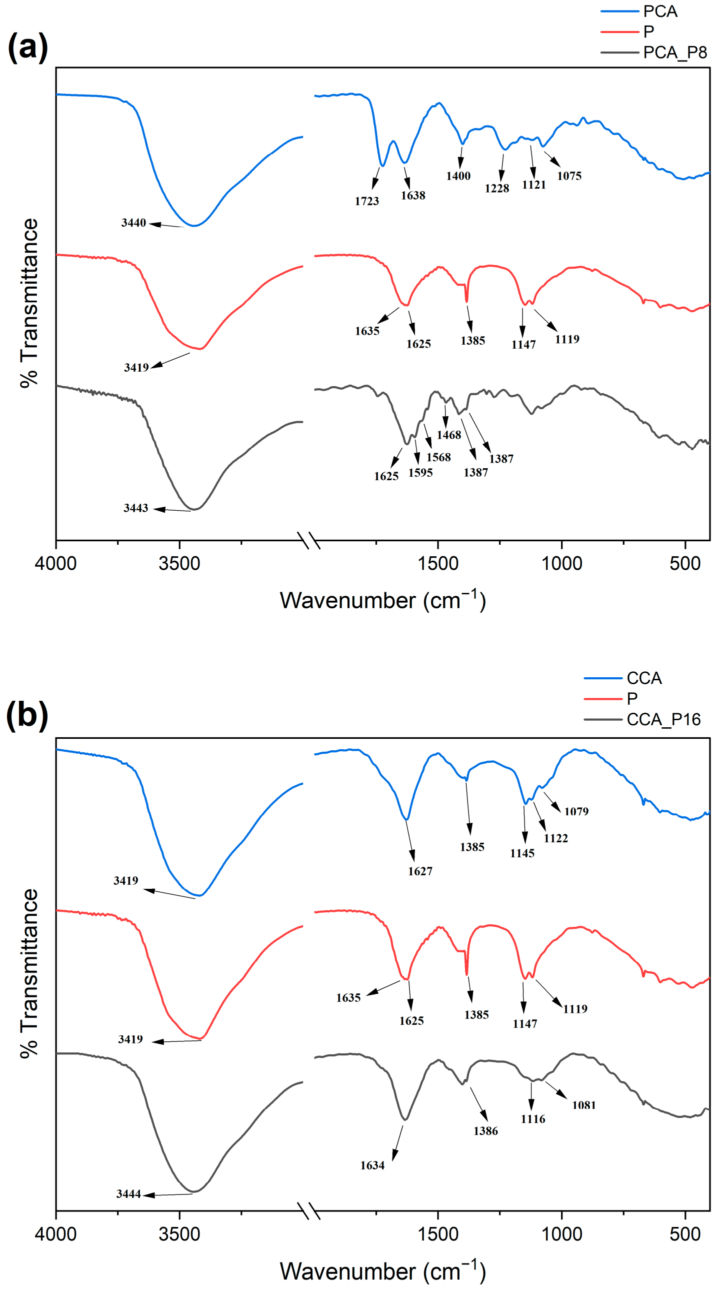

Figure S6. FTIR spectra of copigmented anthocyanins with polyaspartic acid: (a) FTIR spectra of PCA, P and PCA_P8; (b) FTIR spectra of CCA, P, CCA_P16 and CCA_P16.

The FTIR spectrum of C (Figure S7) exhibited characteristic absorption bands at 3454 cm⁻¹ (O–H stretching), 1702 cm⁻¹ (C=O stretching), 1633 cm⁻¹ (olefinic C=C stretching), 1450 cm⁻¹, 1415 cm⁻¹, and 1220 cm⁻¹ (aromatic ring C=C stretching), and 1284 cm⁻¹ (C–O stretching) [66,67]. Significant spectral modifications were observed in copigmented sasmples: in samples PCA_C80 and CCA_C80, the O–H stretching band shifted to lower

wavenumbers (3441 cm^{-1} and 3447 cm^{-1}, respectively) compared to pure C, indicating hydrogen bond formation between the anthocyanins and C. Similarly, the C=O stretching band of C (1702 cm^{-1}) shifted to 1680 cm^{-1} in PCA_C80 and 1674 cm^{-1} in CCA_C80, further confirming hydrogen bonding interactions. The aromatic C=C stretching band at 1415 cm^{-1} shifted to 1420 cm^{-1} (PCA_C80) and 1421 cm^{-1} (CCA_C80), suggesting π–π stacking between the aromatic rings of C and the anthocyanins. In double copigmentation sasmples (PCA_CP and CCA_CP), the O–H stretching band of C shifted to 3423 cm^{-1} and 3444 cm^{-1}, respectively, while the C=O stretching band shifted to 1684 cm^{-1} in PCA_CP and disappeared entirely in CCA_CP. Notably, no significant changes occurred in the C=C stretching regions compared to single copigmentation samples. These observations demonstrate that the addition of P primarily alters the hydrogen bonding interactions between C and the anthocyanins.

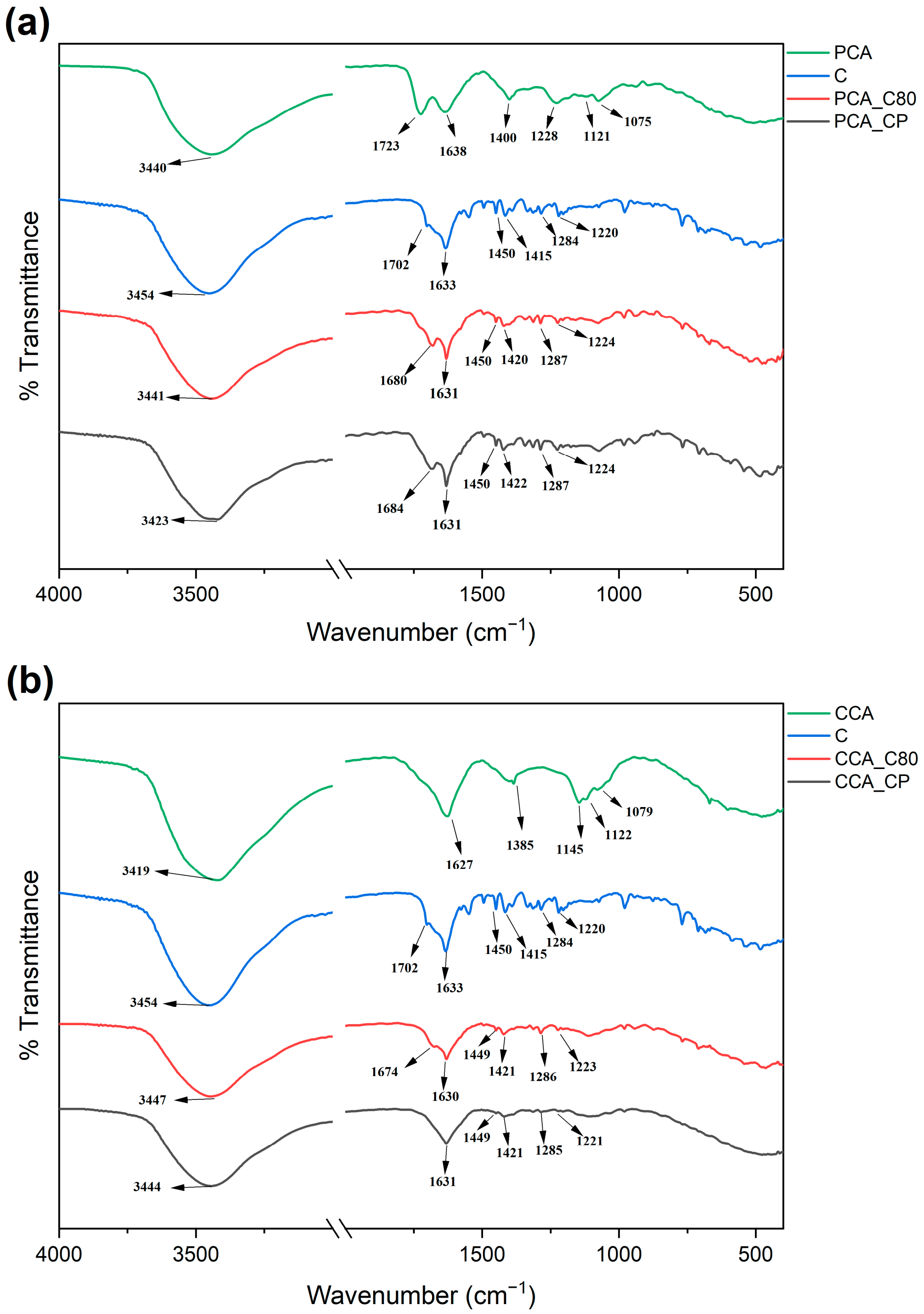

Figure S7. FTIR spectra of copigmented anthocyanins with cinnamic acid: (a) FTIR spectra of PCA, C, PCA_C80 and PCA_CP; (b) FTIR spectra of CCA, C, CCA_C80 and CCA_CP.

The FTIR spectrum of F exhibited characteristic absorption bands at 3439 cm^{-1} (O–H stretching), 1692 cm^{-1} (C=O stretching), 1623 cm^{-1} (olefinic C=C stretching), 1596 cm^{-1} and 1519 cm^{-1} (aromatic C=C stretching), and 1204 cm^{-1} and 1031 cm^{-1} (C–OCH_3 stretching and out-of-plane bending, respectively) [68]. Significant spectral changes were observed upon copigmentation with anthocyanins: in the PCA_F80 sample, the disappearance of the C=O stretching band of PCA indicated preferential stabilization of the flavylium cation over the quinonoid form when complexed with F. The O–H stretching band in CCA_F80 shifted to 3425 cm^{-1} compared to pure F, while the characteristic C=O stretching band of F at 1692 cm^{-1} was nearly absent in both PCA_F80 and CCA_F80. The intensity of the methoxy group vibrations (1204 cm^{-1} and 1031 cm^{-1}) decreased, and the aromatic C=C bands (1596

cm^{-1} and 1519 cm^{-1}) underwent shifts and intensity reductions. These modifications provide evidence of hydrogen bonding and π–π interactions between both PCA and CCA with FA. In the double copigmentation samples (PCA_FP and CCA_FP), further alterations were observed: the O–H stretching bands shifted relative to their single copigment counterparts, and the C=C bands—originally at 1627 cm^{-1} for PCA_F80 and 1621 cm^{-1} for CCA_F80—shifted to 1621 cm^{-1} and 1625 cm^{-1}, respectively. These changes indicate that the addition of P modifies both the hydrogen bonding and hydrophobic interactions between the anthocyanins and F.

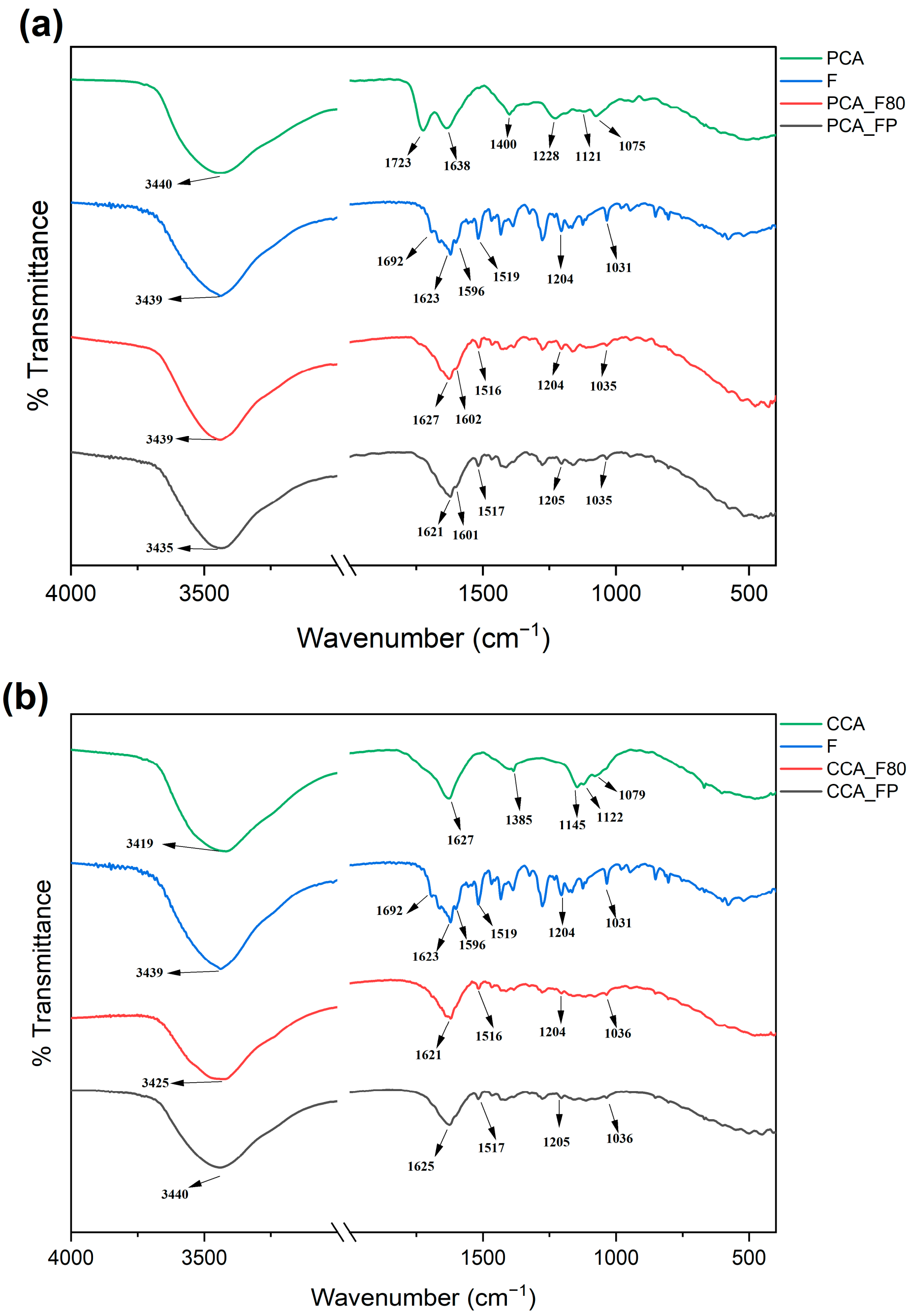

Figure S8. FTIR spectra of copigmented anthocyanins with ferulic acid: (a) FTIR spectra of PCA, F, PCA_F80 and PCA_FP; (b) FTIR spectra of CCA, F, CCA_F80 and CCA_FP.
